# Factors associated with nursing educators’ instructional difficulties in teaching infusion-management skills and students’ infusion-management implementation ability

**DOI:** 10.20407/fmj.2025-018

**Published:** 2026-02-28

**Authors:** Chikako Oda, Sayuri Nakamura, Yumiko Miyoshi, Keiko Onogi

**Affiliations:** Graduate School of Health Sciences, Fujita Health University, Toyoake, Aichi, Japan

**Keywords:** Infusion management, Implementation ability, Nursing students, Instructional difficulties, Associated factors

## Abstract

**Objective:**

To examine the relationship between the difficulty of teaching infusion-management techniques during skills training and clinical practicum phases, the factors influencing this relationship, and the status of students’ ability to implement infusion-management skills (implementation ability), as perceived by educators.

**Methods:**

Educators at three-year basic nursing education institutions nationwide were surveyed and their multiple-choice and open-ended responses to a web survey, which ran from May to September 2023, were analyzed quantitatively and qualitatively.

**Results:**

During the skills training phase, the difficulty of replicating infusion insertion was associated with the difficulty of teaching infusion-management skills acquisition, and the higher the instructional difficulty, the lower the students’ implementation ability. In the clinical practicum stage, the lack of opportunities to practice infusion management, students’ lack of knowledge of it and its necessity were associated with difficulties in teaching infusion management implementation. Additionally, the higher the difficulty of teaching, the lower the student’s implementation ability (under supervision). Moreover, the difficulty of teaching during the skills training phase was also associated with the clinical practicum phase. These instructional difficulties were driven by the lack of reality of educational materials, restrictions on safety and ethical considerations, lack of human resources, and difficulty in coordinating with training facilities.

**Conclusion:**

Difficulties in reproducing educational materials, limited opportunities to implement infusion management, and students’ lack of infusion-management knowledge and skills influenced the difficulty of teaching infusion-management techniques. From an educational perspective, developing educational materials with increased visibility and coordinating with training facilities could help mitigate these challenges.

## Introduction

Infusion management is a common and key component of basic nursing education curricula; thus, it necessitates that students acquire practical skills including observation, assessment, and response, with a focus on intravenous injections.^1^ This is the case not only in Japan, but also in Australia,^2^ the United States,^3^ the United Kingdom,^4^ and elsewhere. Some countries have systems that allow students to administer intravenous injections and conduct infusion management under the supervision of educators or on-site clinical practicum instructors,^5–8^ with some educational institutions identifying it as a goal to be achieved by graduation.^9^

In on-campus skills training (hereinafter “skills training”), theory-based education is offered in conjunction with on-site clinical practicum (hereinafter “clinical practicum”), which includes high-fidelity simulators^10^ and intensive programs,^11^ as well as educational models created through collaboration between educators and clinical practicum instructors.^12^ However, the integration of knowledge and skills, and the acquisition of practical skills, remain challenging endeavors that call for improvements in pedagogy.^4,13,14^ While the use of hybrid and digital simulation is increasing, the recommendation is for such approaches to be used in conjunction with conventional practices.^15,16^

In Japan, accompanying the curriculum revisions from FY2022, review committee reports have divided graduation goals in terms of skills training and clinical practicum, wherein skills training is conducted using model dolls or among students and clinical practicum is conducted clinically. Regarding infusion-management skills, it has become a requirement not only to “perform under supervision in skills training” but also to “perform under supervision in clinical practicum.”^1^ Thus, the aim of skills training is to teach students appropriate procedures and reasons for skills, while that of clinical practicum is to integrate the learned knowledge and skills and help students practice them on actual patients.

Currently, the introduction of simulation education is progressing,^17^ but many schools lack high-performance simulators,^18^ thus limiting skills acquisition. In particular, learning that involves observing and assessing insertion sites may demonstrate creativity in self-made educational materials, but the visibility- and stability-related problems inherent therein make replication difficult.^19,20^ Moreover, difficulties with replicating clinical situations tend to keep students’ understanding abstract.^21^

For example, in adult nursing clinical practicum (acute phase), 3–33% of students performed infusion management, 8–20% of students observed, and 31–80% had no experience.^22–25^ Moreover, a survey of students about to graduate reported that many expressed anxieties about not having been able to conduct clinical practicum, with this lack of learning opportunities affecting their confidence and safety awareness.^26^

Thus, infusion-management skills education involving skills training and clinical practicum is compromised by problems such as limited replicability of educational materials, lack of learning opportunities for students, and instructional difficulties experienced by educators. Despite individual reports confirming these challenges, research that systematically examines these educational issues remains insufficient, causing the sluggish development of concrete countermeasures. As such, testing hypotheses based on a conceptual framework of factors related to the difficulty experienced by instructors in teaching infusion-management skills and the students’ ability to implement their infusion-management skills (students’ implementation ability) is crucial for the development of educational interventions.

Thus, this study examined the following six hypotheses based on a conceptual framework of factors related to instructors’ difficulties in imparting infusion-management skills and students’ ability to implement these skills during the skills training and clinical practicum phases, focusing on infusion-management skills in basic nursing education. The study simultaneously sought to clarify the factors associated with this relationship, as expressed in the following hypotheses.

[Skills training phase]

Hypothesis 1: “Difficulty replicating infusion insertion sites” is a factor associated with “Instructional difficulty in facilitating infusion-management skill acquisition.”

Hypothesis 2: “Instructional difficulty in facilitating infusion-management skill acquisition” is associated with “Students’ ability to implement infusion-management skill items” as perceived by educators.

[Clinical practicum phase]

Hypothesis 3: “Students’ opportunities to observe and perform infusion management” is a factor associated with “Instructional difficulty in implementing infusion management” during the clinical practicum phase.

Hypothesis 4: “Students’ knowledge of infusion management and recognition of its necessity” is a factor associated with “Instructional difficulty in implementing infusion management.”

Hypothesis 5: “Instructional difficulty in implementing infusion management” during the clinical practicum phase is associated with “Proportion of students able to perform infusion management (under supervision).”

[Skills training and clinical practicum phases]

Hypothesis 6: “Instructional difficulty in facilitating infusion-management skill acquisition” during the skills training phase is associated with “Instructional difficulty in implementing infusion management” during the clinical practicum phase.

## Methods

### Terminology

Infusion management refers to the process of observing the remaining infusion volume, drip rate, route connection site, insertion site, and surrounding skin condition to determine and report the situation. Drip adjustment and similar elements are meant to be performed under supervision.

### The study’s conceptual framework

**Figure 1:**
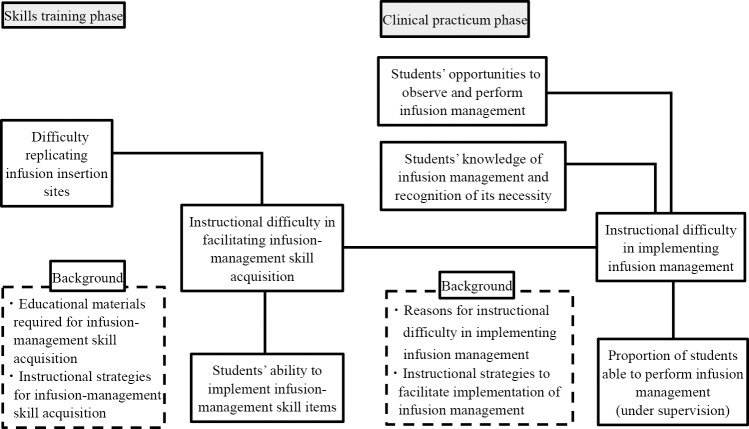
Conceptual framework of factors associated with the relationship between educators’ instructional difficulty and students’ implementation ability in infusion management

This study’s conceptual framework organizes the associated factors potentially forming the background of the relationship between educators’ instructional difficulty and students’ implementation ability during the skills training and clinical practicum phases (Figure 1). For students to properly acquire infusion-management skills, well prepared and equipped educational environments and effective teaching methods are vital. However, issues such as inadequate educational materials and lack of learning opportunities may be present, likely contributing to instructional difficulties and affecting students’ skill acquisition and performance.

During the skills training phase, “Difficulty replicating infusion insertion sites” is framed as a factor associated with “Instructional difficulty in facilitating infusion-management skill acquisition.” Furthermore, an association between “Instructional difficulty in facilitating infusion-management skill acquisition” and “Students’ ability to implement infusion-management skill items” is assumed. These assumptions are based on previous studies that have suggested educators’ inability to adequately replicate clinical scenarios and students’ understanding remaining abstract.^21^ Additionally, the descriptions of “Educational materials required for infusion-management skill acquisition” and “Instructional strategies for infusion-management skill acquisition” were framed as supplementary information to better understand the background of the instructional difficulties.

During the clinical practicum phase, “Students’ opportunities to observe and perform infusion management” and “Students’ knowledge of infusion management and recognition of its necessity” were framed as factors associated with “Instructional difficulty in implementing infusion management.” Furthermore, a relationship between “Instructional difficulty in implementing infusion management” and “Students’ ability to perform infusion management (under supervision)” was assumed. These assumptions were based on previous research that has suggested that students have limited opportunities to practice infusion-management skills, lack of performance experience, and compromised self-confidence and safety awareness.^22–26^ Additionally, the descriptions of “Reasons for instructional difficulty in implementing infusion management” and “Instructional strategies to facilitate implementation of infusion management” were framed as supplementary information to better understand the background of the instructional difficulties.

Moreover, it was assumed that the instructional difficulties experienced during the skills training phase may have also been associated with the instructional difficulties experienced during the clinical practicum phase. This is because, in addition to the conventional requirement to “perform in skills training,” the level of infusion management skills to be achieved by graduation has been raised to “perform also in clinical practicum.”^1^

### Study design

A hypothesis-testing mixed methods study design using an online questionnaire was adopted. More concretely, the previously described six hypotheses concerning the skills training and clinical practicum phases were formulated, associations were verified by quantitative analysis, and background information pertinent to interpreting the quantitative findings through qualitative descriptive analysis was organized.

### Study scope

For participants, the study targeted educators with at least two years of instructional experience in either basic or adult nursing at a three-year basic nursing education institution (hereinafter, “school”) anywhere in Japan, with the aim of enlisting one person per field.

### Duration and methods of the study

The survey period was set for May through September 2023. Written requests (1,400 letters in total) were mailed to school administrators, with participating schools requested to distribute them to potential participants through administrators. Concerning online collection, IP addresses and terminal identification information were used for each field to screen duplicate responses in the same field.

### Survey contents

A self-administered online questionnaire survey was conducted. The survey gathered information on the educators’ basic characteristics (sex, age, type of school, specialization taught, years of teaching experience, and relationships with clinical practicum facilities), the situation regarding skills training and clinical practicum phases, as well as their perceptions of their own students’ situations at their respective institutions (Tables 1 and 2). For the skills training phase, they were asked about “Difficulty replicating infusion insertion sites,” “Instructional difficulty in facilitating infusion-management skill acquisition,” and “Students’ ability to implement infusion-management skill items” according to a five-point scale. Moreover, they were asked to describe educational materials and strategies required for infusion-management skill acquisition through free-form answers. For the clinical practicum phase, they were asked about “Students’ opportunities to observe and perform infusion management,” “Students’ knowledge of infusion management and recognition of its necessity,” “Instructional difficulty in implementing infusion management,” and “Proportion of students able to perform infusion management (under supervision)” according to a five-point scale. Moreover, they were asked to describe reasons for instructional difficulty in and instructional strategies for implementation in free-form answers.

### Analytical methods

A simple aggregation was performed for each question item, calculating distribution, percentage, mean, and standard deviation. Moreover, when checking the distribution, the quantitative items were integrated into two groups: “positive tendency (sufficient/so-so)” and “negative tendency (not much/not at all)” to identify trends. Furthermore, having confirmed response distribution of responses according to the five-point scale (Likert scale) by field (basic nursing/adult nursing) through simple aggregation, it was found that the distributions were generally similar, demonstrating no obvious differences.

Subsequently, the main analysis was conducted by integrating the fields. The analysis made use of non-parametric testing in SPSS version 26, setting the significance level at 5%. The relationship between instructional difficulties and implementation status during each phase was analyzed using Spearman’s rank correlation coefficient. More specifically, for the skills training phase, the analysis concerned the relationship between “Difficulty replicating infusion insertion sites” and “Instructional difficulty in facilitating infusion-management skill acquisition” (Hypothesis 1) as well as that between “Difficulty in teaching infusion-management techniques” and “Students’ ability to implement infusion-management skill items” (Hypothesis 2). Missing values underwent pairwise deletion, with the effective n of each analysis specified in the table.

Analysis the clinical practicum phase examined the relationships between (1) “Students’ opportunities to observe and perform infusion management” and “Instructional difficulty in implementing infusion management” (Hypothesis 3), (2) “Students’ knowledge of infusion management and recognition of its necessity” and “Instructional difficulty in implementing infusion management” (Hypothesis 4), and (3) “Instructional difficulty in implementing infusion management” and “Proportion of students able to perform infusion management (under supervision)” (Hypothesis 5). Moreover, for instructional difficulties during both the skills training phase and the clinical practicum phase, the relationship between “Instructional difficulty in facilitating infusion-management skill acquisition” and “Instructional difficulty in implementing infusion management” (Hypothesis 6) was analyzed. Moreover, for instructional difficulty, it was defined that “the higher the score, the more difficulty experienced,” while for students’ implementation ability, it was defined that “the higher the score, the better the implementation ability.”

Additionally, free-form answers were coded in semantic units by qualitative descriptive analysis and categorized based on similarity and relevance. As part of the analysis, the contents of the descriptions were faithfully handled, and opinions were exchanged among researchers with experience in qualitative research until a consensus was reached.

### Ethical considerations

This study was conducted with the approval of the Medical Research Ethics Review Committee of the university to which the principal investigator belongs (approval number:HM22-195). Written requests were sent out to school administrators stating an overview of the research as well as its purpose, methods and duration, selection criteria, anonymity provisions, respect for free will, and required response time. If consent was obtained, requests were distributed to potential participants via the administrators. The participants responded to the questionnaire survey after confirming the receipt of the consent document online and checking the consent box.

## Results

From the 1,400 written request letters, which were sent in batches of two to 700 schools, 421 responses (response rate 30.1%) were collected. Of these, 375 were included in the analysis after excluding invalid responses (valid response rate: 89.1%). The results are described below, with category names indicated with [ ] and subcategory names indicated with < >.

### Basic characteristics of participants and institutions

As shown in Table 3, the mean age of the participants was 47.9 years (±8.1 years) and the average number of nursing educators’ years of experience was 11.3 years (±7.5 years). Most of the participants (172: 45.9%) specialized in basic nursing, followed by those who specialized in adult nursing (161: 42.9%).

Regarding institutional characteristics, most of the schools the respondents worked at (232: 61.9%) were vocational schools, followed by 122 (32.5%) universities. Concerning the schools’ relationships with clinical practicum facilities, most (239) made requests to clinical practicum hospitals, 176 were in the same organization, and 28 schools had signed cooperation agreements.

### Skills training phase

**Figure 2:**
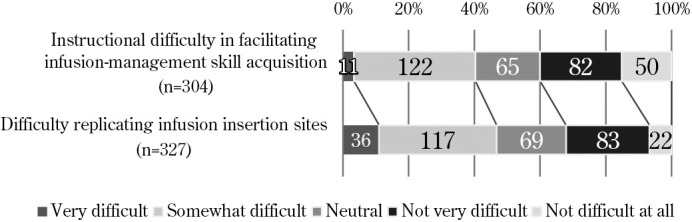
Difficulties in infusion insertion-site replication and infusion-management skill acquisition

**Figure 3:**
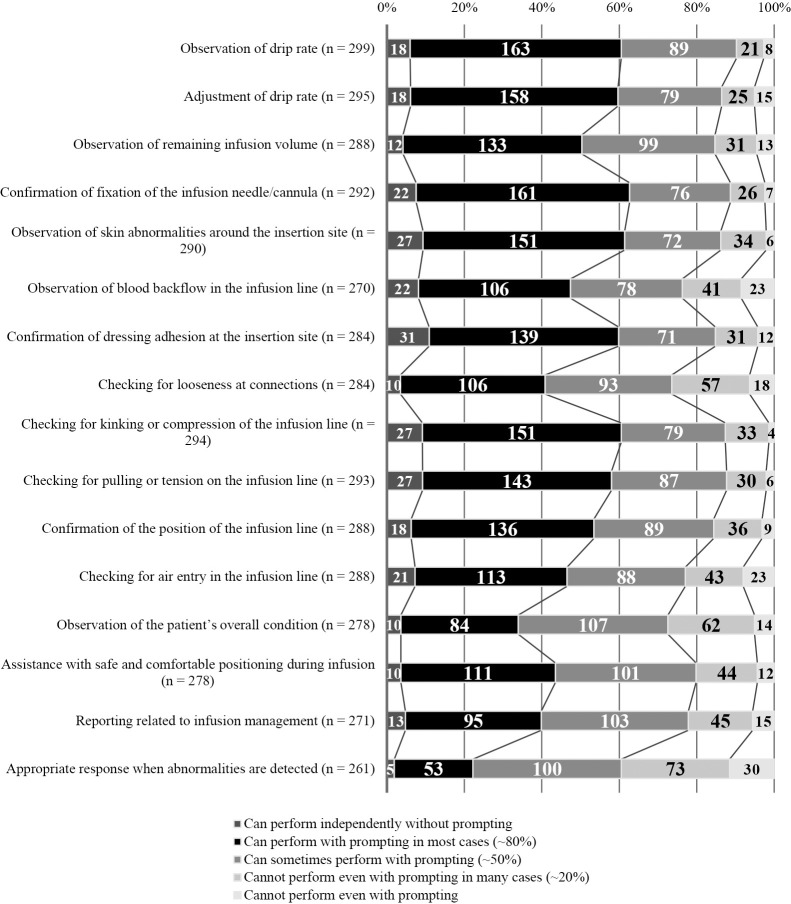
Students’ ability to implement infusion-management skill items

1. Difficulty replicating infusion insertion sites (Figure 2): Of the respondents, 153 (46.7%) answered that it is difficult to replicate infusion insertion sites, while 105 (32.1%) answered that it is not difficult.
2. Instructional difficulty in facilitating infusion-management skill acquisition (Figure 2): Of the respondents, 133 (40.0%) answered that it is difficult to teach the acquisition of infusion management skills, while 132 (40.0%) answered that it is not difficult, indicating polarization.
3. Students’ ability to implement infusion-management skill items (Figure 3): More than 50% of teachers answered that about 80% of the students “Can implement infusion-management with prompting and support in most cases” (~80%) concerning “Observation of drip rate” (52.9%), “Confirmation of secure fixation at the infusion site” (52.3%), and “Adjustment of drip count” (51.3%). Meanwhile, educators answered that, even with advice and prompting, students were able to perform only about half of the required procedures for “Confirmation of air in the infusion route,” “Confirmation of connection site in the infusion route,” and “Response to abnormalities.”
4. Association between difficulty replicating infusion insertion sites and instructional difficulty in facilitating infusion-management skill acquisition (Table 4): This association demonstrated a weak correlation (ρ=0.399, p<0.01).
5. Association between instructional difficulty in facilitating infusion-management skill acquisition and students’ ability to implement infusion-management skill items (Table 5): In this association, a weak negative correlation was found between instructional difficulty and several items, including “Observation of drip rate” (ρ=−.329, p<0.01) and “Adjustment of drip count” (ρ=−.301, p<0.01).
6. Educational materials required for infusion-management skill acquisition (Table 6): The free-form answers respondents provided concerning required educational materials were classified into 8 categories and 23 subcategories, including [Realistic structure simulating the puncture site], [Reproduction of abnormal findings], and [Drip management with possible support].
7. Instructional strategies for infusion-management skill acquisition (Table 7): The respondents’ free-form answers concerning instructional strategies during the skills training phase were classified into 11 categories and 26 subcategories, including [Reproduction of clinical environment], [Promoting critical thinking through questioning and feedback], and [Encouraging situational responses]. Further, several answers described how strategies such as using injection labels and other real items help students engage with the tasks seriously and become aware of the key points of challenges and observations.

### Clinical practicum phase

**Figure 4:**
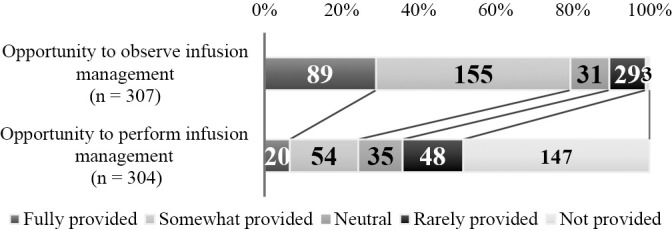
Students’ opportunities to observe and perform infusion management

**Figure 5:**
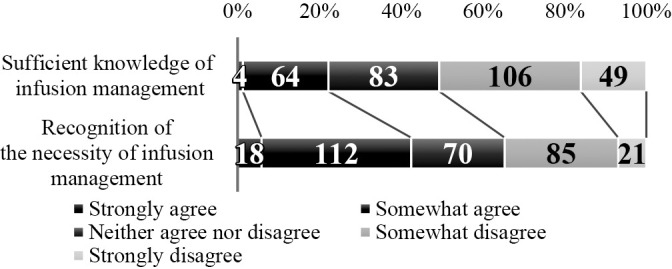
Students’ knowledge of infusion management and recognition of its necessity n=306

**Figure 6:**
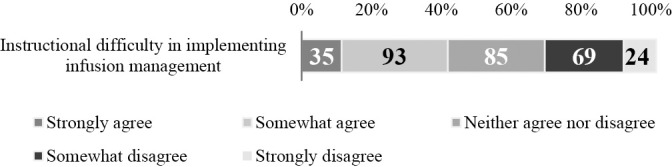
Instructional difficulty in implementing infusion management n=306

**Figure 7:**
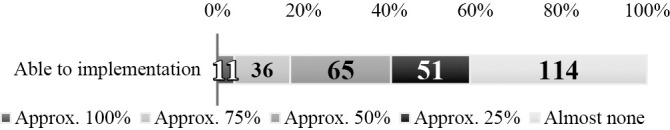
Proportion of students’ able to implementation infusion management (under supervision) n=277

1. Students’ opportunities to observe and perform infusion management (Figure 4): For the clinical practicum, 244 (79.5%) respondents stated that students are given opportunities to observe infusion management, 74 (24.3%) that they are given opportunities to perform it, and 195 (64.1%) that they do not have such opportunities.
2. Students’ knowledge of infusion management and recognition of its necessity (Figure 5): Of the respondents, 68 (22.0%) answered positively that students have “sufficient knowledge” and 155 (50.6%) answered negatively. Regarding whether students recognize the necessity of infusion management, 130 (42.1%) answered positively while 106 (34.3%) answered negatively.
3. Instructional difficulty in implementing infusion management (Figure 6): Of the respondents, 128 (41.8%) answered that it is difficult and 93 (30.3%) answered that it is not difficult.
4. Proportion of students able to implement infusion management (under supervision) (Figure 7): Of the respondents, 47 (17.0%) reported that approximately 100% or 75% of their students could perform infusion management under supervision, while 165 (59.6%) reported that 50% or fewer could perform it under supervision.
5. Association between students’ opportunities to observe and perform infusion management and instructional difficulty in implementing infusion management (Table 8): For this association, weak negative correlations were found for both “Opportunities to observe infusion management” (ρ=–0.260, p<0.01) and “Opportunities to perform infusion management” (ρ=–0.219, p<0.01).
6. Association between students’ knowledge of infusion management and recognition of its necessity and instructional difficulty in implementing infusion management (Table 8): Students’ knowledge of infusion management and recognition of its necessity showed weak negative correlations with instructional difficulty in facilitating infusion-management skill acquisition (knowledge: ρ=–0.207, p<0.01, recognition of necessity: ρ=–0.211, p<0.01).
7. Association between instructional difficulty in implementing infusion management and proportion of students able to perform infusion management (under supervision) (Table 8): The level of students’ ability to implement infusion management under supervision was negatively correlated with instructional difficulty in facilitating infusion-management skill acquisition (ρ=–0.429, p<0.01).
8. Reasons for instructional difficulty in implementing infusion management (Table 9): These were classified into 9 categories and 22 subcategories, including [Insufficient skills enhancement prior to the clinical practicum], [Priority given to safety and ethical considerations], and [Limited capacity due to lack of knowledge and skills].
9. Instructional strategies to facilitate the implementation of infusion management during clinical practicum (Table 10): These were classified into 9 categories and 23 subcategories, including [Promoting experience and observation perspectives in infusion management], [Shared understanding of pre-practicum training content and learning objectives], and [Adjustment of experiential opportunities]. Responses described that [Promoting awareness of observation and assistance during the clinical practicum] and [Reflection and feedback] helped students become aware of and engage actively with infusion management.

### Skills training and clinical practicum phases

Association between instructional difficulty in facilitating infusion-management skill acquisition (skills training phase) and instructional difficulty in implementing infusion management (clinical practicum phase) (Table 11): These were found to be weakly correlated (ρ=0.393, p<0.01).

## Discussion

### The association between instructional difficulty and implementation status during the skills training phase and factors associated with instructional difficulty

Hypothesis 1 was formulated for the skills training phase. The results of the analysis showed that about half of the educators experience difficulty replicating infusion insertion sites, and it was more difficult to facilitate infusion-management skill acquisition. In the free-form answers, a need for realistic educational materials, including [Realistic structure simulating the puncture site], [Reproduction of abnormal findings], and [Drip management with possible support] was also identified.

This need arises because educators recognize that the current educational materials lack the realism necessary for observation and judgment of insertion sites. Thus, replication difficulty may be a factor associated with instructional difficulty. This result is consistent with previous research that has highlighted that support situations where infusion management is performed are difficult to visualize without real images, leading to more reliance on clinical practicum experiences.^21^ Therefore, Hypothesis 1 is supported, indicating that improving replicability in educational materials may reduce the instructional difficulty in facilitating infusion-management skill acquisition.

Regarding Hypothesis 2 the analysis showed that students’ implementation status tends to be so low that educators experience instructional difficulty with regard to skill items. Items that educators recognize as ones that “can be performed with prompting,” such as “Observation of drip rate” and “Adjustment of drip count,” were also found to be associated with instructional difficulties. It is probable that this makes it difficult to facilitate infusion-management skill acquisition, including understanding reasons for procedures and judging the situation. Various strategies were mentioned in the free-form answers, such as <Encouraging judgment in problematic scenarios> under [Encouraging situational responses] and <Confirmation of observation points> under [Promoting critical thinking through questioning and feedback], indicating that educators intentionally provide supervision with reasons and situational judgment.

Previous research has shown that students who do not integrate knowledge and skills are more likely to follow procedures but have difficulty practicing appropriately based on situational judgment and reasons.^6^ Meanwhile, psychological and technical preparation increases students’ self-efficacy,^27^ whereas integration of knowledge and skills contributes to developing students’ judgment.^28^ An association between instructional difficulty in facilitating infusion-management skill acquisition and students’ implementation status of infusion-management skill items was found, thus supporting Hypothesis 2. From an educational point of view, instruction that emphasizes understanding of reasons and situational judgment is preferred.

Thus, difficulty replicating infusion insertion sites is associated with instructional difficulty in facilitating infusion-management skill acquisition, while students’ implementation status for infusion-management skills tends to be so low that educators experience instructional difficulty. This is consistent with previous research that has highlighted that the lack of replicability of clinical situations leads to abstract understanding among students.^21^

In response to such challenges, educators state that they engage in the <Pursuit of realism> using injection labels and other real items for [Reproduction of clinical environment], repeatedly work toward [Acquisition of knowledge and skills], and use questions and feedback for the sake of [Promoting deep and practical thinking]. These methods encourage students to become aware of observation points and make independent efforts. It is suggested that these strategies may help students understand reasons and develop their situational judgment.

However, these strategies in isolation are insufficient to completely counter the fundamental lack of replicability in educational materials and issues with practical understanding. Some reports suggest that developing highly visible educational materials and instruction that assumes clinical situations, such as hybrid simulations and virtual reality (VR), may help improve students’ judgment and confidence.^27,29^ Therefore, a phased introduction will be beneficial.

### The association between instructional difficulties and implementation status during the clinical practicum phase and factors associated with instructional difficulty

Hypothesis 3 was formulated for the clinical practicum phase. The results of the analysis showed that instructional difficulty in implementing infusion management tends to increase when there are fewer opportunities for students to observe and perform infusion management. In particular, about 64% of educators answered that students have no opportunities to perform skills, suggesting a lack of opportunities. The background of relationships with clinical practicum facilities included <High risk makes it difficult to ensure safety> under [Priority given to safety and ethical considerations] and [Coordination difficulties with clinical practicum sites]. Moreover, educational background included <Emphasis on the nursing process> under [Prioritized instruction based on students’ learning phase] and <Educational decision to prioritize basic care> under [Educational policy regarding infusion management]. This background can possibly explain the lack of opportunities to perform skills, causing instructional difficulty. Previous research has reported that many students complete clinical practicum without experiencing infusion management,^22–25^ which is consistent with the results of this study. Thus, Hypothesis 3 is supported. Additionally, opportunities to observe and perform infusion management is found to be associated with instructional difficulty in implementing infusion management during the clinical practicum phase.

For Hypothesis 4 it was found that the lower the knowledge of infusion management and recognition of its necessity, the more likely it is for educators to experience instructional difficulty in implementing infusion management. The free-form answers revealed concrete circumstances such as <Insufficient student knowledge and skills regarding infusion management> and <Students’ low awareness of infusion management> under [Limited capacity due to lack of knowledge and skills]. These are background factors associated with instructional difficulty. The results are consistent with previous research that found that nursing students’ lack of knowledge and skills in infusion management leads to difficulties in implementing infusion management during clinical practicum,^6,7,14^ suggesting the importance of enhancing educational content and practical learning opportunities. Thus, Hypothesis 4 is supported.

Regarding Hypothesis 5 it was found that students’ implementation status of infusion management (under supervision) tends to be lower when educators experience more instructional difficulty. Indicated reasons for instructional difficulty were <Uncertainty in opportunities to care for patients receiving infusions> under [Limited opportunities for hands-on practice] and [Lack of time and human resources for teaching infusion management]. That is, adequate individual supervision is difficult to achieve because it is not easy to secure opportunities where students can be assigned patients subject to infusion management in supervisable situations. This is consistent with reports that it is difficult for students to gain sufficient experience in infusion management, leading to decreased confidence in skills and safety awareness.^22–26^ Moreover, it is consistent with other reports that individual supervision and feedback cannot be sufficiently provided to students due to the burden on educators and a lack of human resources, which is an important factor that exacerbates instructional difficulty.^30,31^ It is suggested that such constraints on the instructional environment may cause instructional difficulty, resulting in a drop in students’ acquisition of infusion-management skills. Thus, Hypothesis 5 is supported.

From the above results pertaining to Hypotheses 3, 4, and 5, it is clarified that instructional difficulty in implementing infusion management during the clinical practicum phase is associated with implementation status under supervision. It is also suggested that constraints associated with ensuring safety and ethical considerations as well as a lack of instruction time and human resources may be the reason for these factors. Accordingly, it may be helpful to align the observation perspective with the criteria for feasibility in advance by sharing learning goals.

### The association between instructional difficulty during the skills training phase and instructional difficulty in the clinical practicum phase

The analysis pertaining to Hypothesis 6 showed that instructional difficulty experienced during the skills training phase is associated with instructional difficulty in implementing infusion management during the clinical practicum phase. The free-form answers listed reasons for instructional difficulty during the clinical practicum phase, including <Lack of educational suitability of instructional materials> and <Difficulty in preparing teaching materials and equipment> under [Insufficient skills enhancement prior to the clinical practicum]. These issues during the skills training phase possibly spill over to the clinical practicum phase as instructional difficulty in implementing infusion management.

In response to such challenges, educators implement instructional strategies such as <Skills check> under [Pre-practicum awareness-raising and skills preparation for infusion management] and [Promoting experience and observation perspectives in infusion management]. Furthermore, there were several responses saying that incorporating “Promoting experience and observation perspectives in infusion management” and “Reflection and feedback” helped students become aware of and engage actively with infusion management. These findings from the free-form answers are consistent with reports that confidence formed during on-campus skills training can support performance during clinical practicum^22^ as well as previous research showing that simulations can improve ability and confidence.^11,32^ The free-form answers suggested that students are aware of and engage actively with infusion management. This finding indicates that acquiring skills and developing self-confidence during the skills training phase may contribute to reducing instructional difficulty during the clinical practicum phase.

### Challenges and solutions in infusion management skills education

It is suggested that difficulty replicating infusion insertion sites, students’ underdeveloped infusion management skills based on situational judgment, lack of opportunities to perform infusion management, and lack of students’ knowledge and recognition of necessity lead to instructional difficulty in infusion management skill education. Previous research has reported that the introduction of simulation materials is an effective way to improve students’ observational skills and clinical judgment.^33,34^ while also demonstrating the importance of practical pedagogy, including questioning and feedback.^28,29^ In terms of concrete measures, it will be effective to introduce practical pedagogy such as highly visible educational materials and VR educational materials as well as scenario-based education during the skills training phase.

During the clinical practicum phase, it is important to coordinate with practicum facilities and strengthen the supervision system. Sharing the students’ implementation status and instruction manuals during the skills training phase with on-site clinical practicum instructors can help clarify the students’ situation and make supervision more effective. It has also been reported that partnerships with clinical practicum facilities support students’ skill acquisition,^35^ thus, ongoing collaboration is necessary. Enhancing students’ clinical inference skills and practical reasoning through strategic instruction^36^ as well as improving the quality of nursing interventions through psychological and technical preparation will also be effective.^37,38^ Therefore, developing skills training programs that emphasize highly visible educational materials and phased development of judgment in preparation for supervised infusion management during the clinical practicum is necessary.

## Conclusion

Difficulties with the replicability of educational materials, limited opportunities to perform infusion management, and a lack of knowledge and skills among students are some of the factors associated with instructional difficulty in infusion management skills. This difficulty during the skills training phase was associated with students’ implementation status of infusion management skill items, whereas during the clinical practicum phase it was associated with students’ implementation status of supervised infusion management. Furthermore, an association was observed between instructional difficulty during the skills training phase and instructional difficulty during the clinical practicum phase.

### Study limitations and future topics

The study makes important contributions to the study of infusion management. However, it had certain limitations. This study was conducted with the participation of educators from AY2023 based on a revision of the level of infusion management skills to be achieved by graduation to “perform under supervision.” The responses do not necessarily reflect consideration of this new goal. Moreover, this study relies on educators’ self-reported answers at a single point in time, thus, there is a risk of bias stemming from the measurement method (common source bias). Moreover, as school IDs were not collected, no adjustments were made at the institutional level, which means that some impact of multiple responses from the same institution cannot be completely ruled out. Moreover, the response rate was low at 30%, thus limiting the generalizability of the findings.

In the future, it will be necessary to expand the number of participants, collect data from both educators and students, and examine the topic from multiple angles to verify generalizability. Furthermore, in addition to the design and trialing of phased skills training, consideration should be given to how coordination is carried out with clinical practicum facilities, including sharing of learning goals, verifying the effectiveness of measures toward the goal of students being able to “perform under supervision in clinical practicum.”

## Figures and Tables

**Table 1:** Survey content—Skills training phase

Survey items (variables)	Measure description	Response format
Difficulty replicating infusion insertion sites	Degree of difficulty in replicating the infusion insertion site using training materials/models	5-point scale: 5=difficult; 1=not difficult
Instructional difficulty in facilitating infusion-management skill acquisition	Degree of instructional difficulty in advancing students to the “able to perform under supervision (using a mannequin or peer practice)” level	5-point scale: 5=difficult; 1=not difficult
Students’ ability to implement infusion-management skill items (16 items)	Extent to which students can currently apply the 16 infusion-management skill items defined with reference to textbooks	5-point scale: 5=can perform independently; 1=unable even with prompting
Educational materials required for infusion-management skill acquisition	Educational materials considered necessary for infusion-management skill acquisition	Free-form response
Instructional strategies for infusion-management skill acquisition	Instructional strategies used to facilitate infusion-management skill acquisition	Free-form response

**Table 2:** Survey content—Clinical practicum phase

Survey items (variables)	Measure description	Response format
Students’ opportunities to observe and perform infusion management	Adequacy of students’ opportunities to observe and perform infusion management during the clinical practicum	5-point scale: 5=ample opportunities; 1=no opportunities
Students’ knowledge of infusion management and recognition of its necessity	Degree to which students are perceived to possess knowledge of infusion management and to recognize its necessity	5-point scale: 5=high; 1=low
Instructional difficulty in implementing infusion management	Degree of instructional difficulty in guiding students to implement infusion management under supervision	5-point scale: 5=difficult; 1=not difficult
Proportion of students able to perform infusion management (under supervision)	Percentage of students who can perform infusion management under supervision at the time of the respondent’s practicum supervision	5-point scale (percentage bands): 5 ≈ 100%; 1=almost none
Reasons for instructional difficulty in implementing infusion management	Reasons for the above instructional difficulties in implementing infusion management	Free-form response
Instructional strategies to facilitate implementation of infusion management	Instructional strategies to enable students to implement infusion management under supervision	Free-form response

**Table 3:** Basic characteristics of participants and institutions (n=375)

	Item	n (%)
Basic Attributes of Participants		
Age Group	20s–30s	59 (15.7)
	40s	155 (41.3)
	50s	135 (36.0)
	60s	26 (6.9)
		Mean age±SD=47.9±8.1 years
Gender	Male	57 (15.2)
	Female	312 (83.2)
	No response	6 (1.6)
Years of Experience as a Nursing Educator	Less than 5 years	74 (19.7)
	5–10 years	115 (30.7)
	10–15 years	75 (20.0)
	15–20 years	48 (12.8)
	20+ years	63 (16.8)
		Mean years±SD=11.3±7.5 years
Area of Expertise	Basic Nursing	172 (45.9)
	Adult Nursing	161 (42.9)
	Integrated Nursing and Practice	8 (2.1)
	Home Care Nursing	9 (2.4)
	Pediatric Nursing	8 (2.1)
	Psychiatric Nursing	4 (1.1)
	Maternal Nursing	6 (1.6)
	Geriatric Nursing	7 (1.9)
Basic Attributes of Organizations		
Type of School	University	122 (32.5)
	Junior College	8 (2.1)
	College of Nursing	3 (0.8)
	Vocational School	232 (61.9)
	Other/No response	10 (2.6)
Relationship with clinical practicum facilities (multiple responses allowed)	Same organization	176
	Educational/research collaboration	28
	Commissioned as clinical practicum facilities	239
	Undetermined	4

**Table 4:** Association between difficulty replicating infusion insertion sites and instructional difficulty in facilitating infusion-management skill acquisition

	Difficulty replicating infusion insertion sites
Instructional difficulty in facilitating infusion-management skill acquisition	0.399**

Spearman’s rank correlation coefficient, ρ; **p<0.01; n=299

**Table 5:** Association between instructional difficulty in facilitating infusion-management skill acquisition and students’ ability to implement infusion-management skill items

	Instructional difficulty in facilitating infusion-management skill acquisition
Observation of drip rate	−0.329**
Adjustment of drip rate	−0.301**
Observation of remaining infusion volume	−0.256**
Confirmation of fixation of the infusion needle/cannula	−0.247**
Observation of skin abnormalities around the insertion site	−0.217**
Observation of blood backflow in the infusion line	−0.164**
Confirmation of dressing adhesion at the insertion site	−0.223**
Checking for looseness at connections	−0.239**
Checking for kinking or compression of the infusion line	−0.239**
Checking for pulling or tension on the infusion line	−0.197**
Confirmation of the position of the infusion line	−0.212**
Checking for air entry in the infusion line	−0.248**
Observation of the patient’s overall condition	−0.165**
Assistance with safe and comfortable positioning during infusion	−0.200**
Reporting related to infusion management	−0.231**
Appropriate response when abnormalities are detected	−0.161**

Spearman’s rank correlation coefficient, ρ; **p<0.01; n=305

**Table 6:** Educational materials required for infusion-management skill acquisition

Category	Sub-category
Realistic structure simulating the puncture site	Materials for confirming blood backflow
	Improved models for observing puncture site and drip
	Materials for observing infusion puncture sites
	Realistic representation of infusion puncture sites
Reproduction of abnormal findings	Materials that simulate complications
	Models that allow observation of abnormal puncture sites
	Models that realistically replicate puncture site abnormalities
Drip management with support	Materials allowing observation of drips
	Materials that enable observation and assistance during dripping
Handling of sequential procedures	Models allowing IV insertion and dripping
	Models allowing venipuncture and confirmation of blood backflow
	Models with tape fixation capability for puncture sites
	Materials for practicing IV puncture
	Pediatric practice models for infusion
Materials with adhesion and fixation	Models with improved tape adhesion
Cost-effectiveness	Affordable training materials
	Low-cost and effective materials
Ease of maintenance	Models with replaceable or reusable parts
	Washable and detachable models
	Easy-to-maintain materials
	Training kits for infusion management
ICT-based learning support	ICT-based individualized learning materials
	Audiovisual learning materials

**Table 7:** Instructional strategies for infusion-management skill acquisition

Category	Sub-category
Pre-learning to confirm knowledge and skills	Pre-learning using audiovisual materials
	Pre-learning for technical training
	Confirmation of knowledge based on rationale
Supervisory framework ensuring student safety and reassurance	Supervisory framework ensuring student safety
	Instruction system designed to reduce fear and anxiety
Instructors acting as simulated patients	Instructors acting as simulated patients
Reproduction of clinical environment	Pursuit of realism
	Use of real or realistic materials
Promoting critical thinking through questioning and feedback	Feedback from instructors and simulated patients
	Confirmation of observation points
Promoting deep and practical thinking	Instruction linked to skills and assessment
	Opportunities for reflection through scenario presentation
	Stepwise acquisition of knowledge and skills
Encouraging situational responses	Encouraging judgment in problematic scenarios
	Experience of care involving patient movement
Experiencing specific roles	Experience of being a patient
	Understanding nurses’ perspectives
Acquisition of knowledge and skills	Acquisition of appropriate techniques
	Learning methods linked to knowledge and skills
	Setting up environments that promote active knowledge and skills
	Use of skill checklists and tests
	Practice of sequential tasks from drip start to finish
Integration and application of knowledge	Connection to the curriculum
	Pre-graduation skills training
	Opportunities to accumulate experience
Pump management	Instruction on operation and management of pumps

**Table 8:** Associations between instructional difficulty in implementing infusion management and related factors

	Instructional difficulty in implementing infusion management
Students’ opportunities to observe infusion management	−0.260** (n=306)
Students’ opportunities to perform infusion management	−0.219** (n=303)
Students’ knowledge of infusion management	−0.207** (n=305)
Students’ recognition of the necessity of infusion management	−0.211** (n=305)
Proportion of students able to perform infusion management (under supervision)	−0.429** (n=277)

Spearman’s rank correlation coefficient, ρ; **p<0.01; pairwise deletion; n varies by pair

**Table 9:** Reasons for instructional difficulty in implementing infusion management

Category	Sub-category
Insufficient skills enhancement prior to the clinical practicum	Lack of educational suitability of instructional materials
	Difficulty in preparing teaching materials and equipment
	Emphasis on other educational content
Prioritized instruction based on students’ learning phase	Emphasis on the nursing process
	Educational phase of the fundamental nursing practicum
Educational policy regarding infusion management	Educational decision to prioritize basic care
	Educational decision considering institutional and ethical constraints
	Outside the scope of educational achievement goals
Limited capacity due to lack of knowledge and skills	Students’ low awareness of infusion management
	Students’ low prioritization of infusion management
	Insufficient student knowledge and skills regarding infusion management
	Students are preoccupied with patient assessment and care
Lack of time and human resources for teaching infusion management	Difficulty in securing instructional time
	Shortage of human resources
Priority given to safety and ethical considerations	High risk makes it difficult to ensure safety
	Ethical considerations for patients take priority
Coordination difficulties with clinical practicum sites	Difficulties coordinating with clinical practicum sites
	Adherence to facility policies
Limited opportunities for hands-on practice	Uncertainty in opportunities to care for patients receiving infusions
	Lack of opportunities to gain experience during the practicum period
	Restrictions on the clinical practicum due to COVID-19
Observation only (no hands-on practice)	Observation only; hands-on practice not allowed

**Table 10:** Instructional strategies to facilitate implementation of infusion management

Category	Sub-category
Pre-practicum awareness-raising and skills preparation for infusion management	Explanation of infusion management during orientation
	Pre-practicum skills check
	Pre-practicum preparatory learning
	Instruction to integrate into care planning
	Promotion of active learning using checklists
Promoting awareness of observation and assistance during the clinical practicum	Encouraging focus on infusion before observation or assistance
	Promotion of active participation
Promoting experience and observation perspectives in infusion management	Thorough instruction in observation and drip rate calculation
	Instruction to raise awareness of infusion content and observation perspectives
Promoting experience within the feasible scope during the clinical practicum	Awareness-building of drip rate calculation as part of infusion management
	Instruction on observation as part of infusion management
	Performing infusion management together with the instructor
	Opportunities to experience infusion management outside assigned patients
Reflection and feedback	Post-observation reflection and prediction
	Promoting understanding through timely reflection
	On-campus practice based on knowledge and experience gained during the clinical practicum
Explanation provided by the instructor	Requesting explanations or guidance from instructors
	Promoting visualization of potential complications
Shared understanding of pre-practicum training content and learning objectives	Understanding of on-campus skills training content
	Achieving a shared understanding of pre-practicum learning content and graduation-level learning objectives through coordination meetings
	Coordinating opportunities for hands-on practice during the clinical practicum through meetings
Adjustment of experiential opportunities	Adjusting experiential opportunities related to infusion management
Adjustment of observation opportunities	Adjustment of observation opportunities

**Table 11:** Association between instructional difficulty in facilitating infusion-management skill acquisition (skills training phase) and instructional difficulty in implementing infusion management (clinical practicum phase)

	Instructional difficulty in implementing infusion management
Instructional difficulty in facilitating infusion-management skill acquisition	0.393**

Spearman’s rank correlation coefficient, ρ; **p<0.01; n=303

## Supplementary Material

PDF-Japanese
