# Supplementary material for: Factors associated with nursing educators’ instructional difficulties in teaching infusion-management skills and students’ infusion-management implementation ability
Source: Fujita Med J. 2026 Feb 28;12(2):159–71. doi: 10.20407/fmj.2025-018 (PMC13129711; doi:10.20407/fmj.2025-018)
Supplement: Supplementary file 1 — PDF-Japanese [file fmj-12-159_s1.pdf]

【タイトル】

輸液管理技術に関する教員の指導困難と学生の輸液管理実施状況の関連要因

【ランニングタイトル】

教員の指導上の困難と学生の輸液管理実施状況

【Authors (Full names, Degrees, Affiliation)】

Chikako Oda, RN, MSN
Graduate School of Health Sciences, Fujita Health University, Toyoake, Aichi, Japan
Sayuri Nakamura, Ph.D.
Graduate School of Health Sciences, Fujita Health University, Toyoake, Aichi, Japan
Yumiko Miyoshi, Ph.D.
Graduate School of Health Sciences, Fujita Health University, Toyoake, Aichi, Japan
Keiko Onogi, M.D., Ph.D.
Graduate School of Health Sciences, Fujita Health University, Toyoake, Aichi, Japan

【Type of Manuscript】

Original Article
Running Title
Educators' Difficulties and Students' Infusion Ability

【Corresponding Author】

Sayuri Nakamura, Ph.D.

【Contact Information】

Graduate School of Health Sciences, Fujita Health University, 1-98 Dengakugakubo, Kutsukake-cho, Toyoake, Aichi 470-1192, Japan
Phone: +81-562-93-9077
Email: sayuri@fujita-hu.ac.jp

抄録
目的：演習・実習において輸液管理技術の指導困難と教員が認識する学生の実施状況との関連を検証し、その解釈に資する背景を明らかにすることで、教育改善への示唆を得る。
方法：2023年度に全国の三年課程看護基礎教育機関の教員を対象とし、Web調査で選択式・自由記述による回答を回収し、量的・質的に分析した。
結果：演習段階では、輸液刺入部の再現性の困難が輸液管理技術習得の指導困難と関連し、指導困難が高いほど学生の輸液管理技術の実施状況が低かった。実習段階では、輸液管理実施機会の不足や学生の知識・必要性の認識不足が輸液管理実施の指導困難と関連し、指導困難が高いほど学生の輸液管理実施状況(指導下)が低かった。また、演習段階の指導困難は実習段階とも関連していた。自由記述からは、教材のリアリティ不足、安全確保や倫理的配慮の制約、人的資源不足、実習施設との調整困難が指導困難の具体的な背景として示された。
結論：教材の再現性困難、輸液管理実施機会の制約、学生の知識・技術不足などが、輸液管理技術に関する指導困難の関連要因であることが示唆された。教育的には，視認性を高めた教材開発、実習施設との目標共有が有用と考えられる。

キーワード
輸液管理、実施状況、看護学生、指導困難、関連要因

序論

看護基礎教育において、輸液管理は重要な教育内容のひとつであり、静脈内注射を中心に、観察・判断・対応を含む実践力の習得が求められている[1]。この傾向は日本のみならず、オーストラリア[2]、アメリカ[3]、イギリス[4]などのカリキュラムにも共通してみられる。一部の国では、教員や臨地実習指導者の監督下で学生が静脈内注射や輸液管理を実施できる制度があり、[5~8]卒業時到達目標として位置付けている教育機関も存在する[9]。

学内演習(以下、演習)では、高忠実度シミュレータ[10]や集中型プログラム[11]など、臨地実習(以下、実習)と連動した教育が行われている。教員と臨地実習指導者の協働による教育モデルの構築もみられる[12]。一方で、知識と技術の統合、実践スキルの習得には課題があり、教育法の改善が求められている[4,13,14]。ハイブリッド型やデジタルシミュレーションの活用も進む一方で、従来型との併用が推奨されている[15,16]。

日本では2022年度からのカリキュラム改正に伴い、検討会報告書では、卒業時の到達目標を演習・実習に区分し、演習はモデル人形または学生間での実施、実習は臨床での実施として整理している。輸液管理技術においては、「演習で指導の下で実施」に加えて「実習で指導の下で実施」も求められるようになった[1]。このように演習では、技術の適切な手順や根拠など習得することを目的とし、実習では、実際の患者を対象に知識・技術を統合して実践することを目的としている。

現在、シミュレーション教育の導入も進んでいるが、[17]高機能シミュレータを所有しない学校も多く[18]技術習得には限界がある。特に刺入部の観察や判断を伴う学習では、自作教材

の工夫もみられるが、視認性や安定性に課題があり再現は容易でない[19,20]。さらに、臨床場面の再現が困難なため、学生の理解が抽象的にとどまる傾向がある[21]。

たとえば成人看護学実習(急性期)では、輸液管理を実施した学生は3～33%、見学は8～20%、未経験は31～80%と高率を占めていた[22~25]。また、卒業間近の学生を対象とした調査では、実習で実施できなかったことへの不安が多く挙げられ、学習機会の不足が学生の自信や安全意識に影響していると報告されている[26]。

このように、演習・実習を通じて輸液管理技術教育には、教材の再現性限界、学習機会の不足、指導困難などの課題がある。学習機会の実態、教員が直面する指導困難については、個別の報告はあるものの、それらを教育上の課題として体系的に整理した研究は蓄積されておらず、具体的な対応策の整備はなお不十分である。したがって、教員の指導困難と学生の実施状況との関連要因について概念枠組みに基づく仮説を検証することには、教育的介入設計に資する意義がある。本研究は、仮説検証から得た知見に基づき、視認性の高い教材の整備や判断力育成を重視した段階的な演習プログラムの設計指針を提案し、「実習で指導の下で実施」[1]の達成に向けた効果的な輸液管理技術教育の基盤構築を目指す。

本研究の目的は、看護基礎教育における輸液管理技術を対象に、演習段階および実習段階において教員の指導困難と教員が認識する学生の輸液管理技術の実施状況との関連要因について、概念枠組みに基づく以下の6つの仮説を検証することである。併せて、仮説の解釈に資する背景も明らかにする。

【演習段階】

仮説1：「輸液刺入部の再現性の困難」は、「輸液管理技術習得の指導困難」に関連する要因である。

仮説2：「輸液管理技術習得の指導困難」は、教員が認識する「学生の輸液管理技術項目実施状況」と関連する。

【実習段階】

仮説3：「学生の輸液管理見学・実施の機会」は、実習段階における「輸液管理実施の指導困難」に関連する要因である。

仮説4：「学生の輸液管理に関する知識・必要性の認識」は、「輸液管理実施の指導困難」と関連する要因である。

仮説5：実習段階における「輸液管理実施の指導困難」は、学生の「学生の輸液管理実施状況(指導下)」に関連する。

【演習段階と実習段階】

仮説6：演習段階での「輸液管理技術習得の指導困難」は、実習段階の「輸液管理実施の指導困難」と関連する。

方法

用語の定義

輸液管理：輸液の残量、滴下速度、ルートの接続部、刺入部や周囲の皮膚の状態などを観察し状況を判断・報告する。滴下調整などは指導下で行うことを指す。

研究の概念枠組み(Figure 1)

本研究の概念枠組みは、演習段階と実習段階において、教員の指導困難と教員が認識する学生の実施状況の関連を中核に、その背景となり得る関連要因を整理したものである。看護基礎教育において、学生が輸液管理技術を適切に習得するためには、教育環境や効果的な教育方法の整備が必要である。しかし、教材整備の不備や学習機会の不足といった課題が存在し、これらが指導困難や学生の技術実施状況と関連し得ると想定する。

演習段階では、「輸液刺入部の再現性の困難」を「輸液管理技術習得の指導困難」に関連する要因として位置づける。さらに、「輸液管理技術習得の指導困難」と「学生の輸液管理技術項目実施状況」の関連を想定する。これらは、臨床場面を十分に再現できず、学生の理解が抽象的に留まる傾向がある[21]先行研究に基づく。加えて、「輸液管理技術習得に必要な教材」および「輸液管理技術習得のための工夫」に関する記述は、指導困難が生じる背景を理解するための補足的な情報として位置づけた。

実習段階では、「学生の輸液管理見学・実施の機会」および「学生の輸液管理に関する知識・必要性の認識」を「輸液管理実施の指導困難」と関連する要因として位置づける。さらに、「輸液管理実施の指導困難」と「学生の輸液管理実施状況(指導下)」の関連を想定する。これらは、学生が輸液管理技術を実施する機会が限られ、実施経験が不足し、自信や安全意識にも影響を及ぼしている[22~26]先行研究に基づく。加えて、「輸液管理実施の指導困難の理由」および「輸液管理実施のための工夫」に関する記述は、指導困難が生じる背景を理解するための補足的な情報として位置づけた。

また、演習段階の指導困難は、実習段階の指導困難とも関連し得ると想定する。これは、輸液管理技術の卒業時到達水準が従来の「演習で実施」に加え、「実習でも実施」することへと引き上げられた[1]ことで、演習段階における指導困難が実習段階での指導困難に関連する可能性が高まったと考えた。

研究デザイン

Web アンケートを用いた仮説検証型の混合研究デザインを採用した。具体的には、研究目的で掲げた演習段階および実習段階における 6 つの仮説を設定し、量的分析で関連を検証し、質的記述的分析で量的所見の解釈に資する背景を整理した。

研究対象

全国の三年課程看護基礎教育機関(以下、学校)に所属し、基礎看護学、成人看護学の分野で指導経験が 2 年以上ある教員とした。各分野 1 名を想定した。

調査期間と方法

調査期間は、2023 年 5 月～9 月とした。学校管理者へ依頼文書を郵送し、承諾校は管理者経由で、基礎看護学および成人看護学の研究対象者に配布を依頼した。Web 回収では、分野ごとに IP アドレスと端末識別情報を用いて、同一分野の重複回答をスクリーニングした。

調査内容(Table 1, Table 2)

自施設における担当分野の学生の状況について、教員の認識に基づく自己記入形式Webアンケートとした。対象者の基本属性(担当専門分野、教員経験年数、性別、年齢、学校の種別、実習施設との関連)に加え、演習段階と実習段階の状況を尋ねた。演習段階では「輸液刺入部の再現性の困難」「輸液管理技術習得の指導困難」「学生の輸液管理技術項目実施状況」を5件法で尋ねた。また、輸液管理技術習得に必要な教材と工夫を自由記述で収集した。実習段階では、「学生の輸液管理の見学・実施機会」「学生の輸液管理に関する知識・必要性の認識」「輸液管理実施の指導困難」「学生の輸液管理実施状況(指導下)」を5件法で尋ねた。さらに、実施の指導困難の理由と実施のための工夫を自由記述で収集した。

分析方法

(1)各質問項目について単純集計を行い、分布、百分率、平均値、標準偏差を算出した。また、分布の確認にあたって、量的項目は「肯定的傾向(十分・まあまあ)」「否定的傾向(あまり・全く)」の2群に統合し、その傾向を把握した。さらに、分野(基礎看護学／成人看護学)別に5件法(Likert尺度)の回答分布を単純集計で確認したところ、分布は概ね類似しており、明確な差異はみられなかった。

(2) 上記の結果に基づき、主要分析は分野を統合して実施した。SPSS version 26を用いてノンパラメトリック検定で分析し、有意水準を5%とした。各段階における指導困難と実施状況の関連をSpearmanの順位相関係数を用いて分析した。具体的には、演習段階で「輸液刺入部の再現性の困難」と「輸液管理技術習得の指導困難」(仮説1)、「輸液管理技術習得の指導困難」と「学生の輸液管理技術項目実施状況」(仮説2)の関連を分析した。欠測はペアワイズ削除とし、各分析の有効nを表中に明記した。

実習段階では、「学生の輸液管理見学・実施の機会」と「輸液管理実施の指導困難」(仮説3)、「輸液管理に関する知識や必要性の認識」と「輸液管理実施の指導困難」(仮説4)、「輸液管理実施の指導困難」と「学生の輸液管理実施状況(指導下)」(仮説5)の関連を分析した。また、演習段階と実習段階の指導困難では、「輸液管理技術習得の指導困難」と「輸液管理実施の指導困難」(仮説6)の関連を分析した。なお、指導困難については「得点が高いほど困難を感じる」、実施状況については「得点が高いほど良好な状況」を示すとした。

(3)自由記述は質的記述的分析により、意味単位ごとにコード化し、類似性・関連性に基づきカテゴリ化した。分析にあたり、記述内容に忠実に向き合い、質的研究の経験者と共に研究者間で合意に至るまで意見交換を行った。

倫理的配慮

研究責任者が所属する大学の医学研究倫理審査委員会の承認(承認番号HM22-195)を得て実施した。学校の管理者宛に研究趣旨、目的、 方法・期間、選定理由、匿名性の配慮、自由意思の尊重、回答所要時間などを記載した依頼文書を送付した。承諾が得られた場合は、

管理者を通じて研究対象者に依頼文書を配布した。対象者は、Web 上の同意文書を確認し、同意欄にチェックを入れたうえでアンケートに回答した。

利益相反

研究責任者が所属する大学の利益相反委員会にて承認(承認番号 CI22-346)を得ており、開示すべき利益相反はない。

結果

依頼文書は 700 校に各 2 通(計 1400 通)に送付した。Web 上で同意のうえ回答した 421 件(回収率 30.1%)を回収し、無効を除く有効回答は 375 件(有効回答率 89.1%)を分析対象とした。以下本文中では、カテゴリ名を【 】、サブカテゴリを＜＞で示す。

参加者および組織の基本属性(Table3)

参加者の平均年齢は 47.9 歳(±8.1 歳)で、看護教員歴の平均は 11.3 年(±7.5 年)であった。専門分野は、基礎看護学 172 人(45.9%)、成人看護学 161 人(42.9%)、看護の統合と実践 8(2.1%)、その他が 34 人(9.1%)であった。

組織の属性として、学校の種別は、専門学校が 232 人(61.9%)、大学 122 人(32.5%)などであった。実習施設との関係では、実習病院として依頼が最多で 239 人、同じ設置主体 176 人、連携協定を結ぶ学校も見られた。

演習段階

①輸液刺入部の再現性の困難(Figure2)

輸液刺入部の再現が困難である回答が 153 人(46.7%)、困難ではない回答が 105 人(32.1%)であった。

②輸液管理技術習得の指導困難(Figure2)

輸液管理技術習得の指導が困難である回答が 133 人(40.0%)、困難ではない回答が 132 人(40.0%)であり、二極化していた。

③学生の輸液管理技術項目実施状況(Figure3)

輸液管理技術項目の実施状況について、「助言や促しがあれば、学生の 8 割程度が実施できる」と教員が回答した割合が 50%を超えたのは、「滴下速度の観察」(52.9%)、「刺入針の固定確認」(52.3%)、「滴下数の調整」(51.3%)であった。一方、「空気混入の確認」「接続部の確認」「異常時の適切な対応」については、助言や促しがあっても助言や促しがあっても、求められる内容の半分程度しか実施できないと認識していた。

④輸液刺入部の再現性の困難と輸液管理技術習得の指導困難の関連(Table4)

輸液刺入部の再現性の困難と輸液管理技術習得の指導困難の関連をみると、$\rho=0.399$、$p<0.01$ と弱い相関が示された。

⑤輸液管理技術習得の指導困難と学生の輸液管理技術項目実施状況の関連(Table5)

輸液管理技術習得の指導困難と学生の輸液管理技術項目実施状況の関連では、「滴下速度の観察」（ρ =-.329, $p<0.01$)、「滴下数の調整」（ρ =-.301, $p<0.01$)など、多くの項目において指導困難との間に弱い負の相関が認められた。

⑥輸液管理技術習得に必要な教材(Table6)

必要な教材に関する自由記述は、【刺入部を再現したリアルな構造】、【リアルな異常所見の再現】【援助が可能な滴下対応】を含む、8のカテゴリと23のサブカテゴリで構成された。

⑦輸液管理技術習得のための工夫(Table7)

演習の工夫に関する自由記述は、【臨床的環境の再現】【発問やフィードバックによる思考の促進】【状況に応じた対応の思考】などを含む、11のカテゴリと26のサブカテゴリで構成された。これらの工夫により、注射ラベルなど実物を使用することで学生が真剣に取り組み、課題や観察の要点を意識化したとの記載が複数みられた。

実習段階

①学生の輸液管理見学・実施の機会(Figure4)

実習において、輸液管理の見学機会が得られる回答は244人(79.5%)、実施機会が得られる回答は74人(24.3%)、得られない回答は195人(64.1%)であった。

②学生の輸液管理に関する知識・必要性の認識(Figure5)

学生の輸液管理に関する知識については、「十分得ている」との肯定的回答が68人(22.0%)、否定的回答が155人(50.6%)であった。学生が輸液管理の必要性を認識できているかについては、肯定的回答が130人(42.1%)、否定的回答が106人(34.3%)であった。

③輸液管理実施の指導困難　(Figure6)

輸液管理実施の指導困難では、困難である回答が128人(41.8%)、困難ではない回答が93人(30.3%)であった。

④学生の輸液管理実施状況(指導下)(Figure7)

学生の輸液管理実施状況(指導下)は、100%と75%程度の合計が47人(17.0%)、50%程度以下の合計は165人(59.6%)であった。

⑤学生の輸液管理見学・実施の機会と輸液管理実施の指導困難の関連(Table8)

輸液管理の見学・実施機会と輸液管理実施の指導困難との関連では、「輸液管理の見学機会」（ρ =-0.260, $p<0.01$)、「輸液管理の実施機会」（ρ =-0.219, $p<0.01$)ともに弱い負の相関が認められた。

⑥学生の輸液管理に関する知識・必要性の認識と輸液管理実施の指導困難の関連(Table8)

学生の輸液管理に関する知識および必要性の認識は、それぞれ輸液管理実施の指導困難と弱い負の相関が認められた(知識：ρ=-0.207, $p<0.01$、必要性の認識：ρ=-0.211, $p<0.01$)。

⑦輸液管理実施の指導困難と学生の輸液管理実施状況(指導下)の関連(Table8)

輸液管理実施の指導困難と学生の輸液管理実施状況(指導下)の関連では、（ρ =-0.429、$p < 0.01$)と負の相関が示された。

⑧輸液管理実施の指導困難の理由(Table9)

【実習前に技術を高める演習が不十分】、【安全確保と倫理的配慮を優先】、【学生の知識・技術不足による余裕のなさ】を含む9のカテゴリと22のサブカテゴリが構成された。

⑨輸液管理の実施に向けた実習での工夫(Table10)

実習において学生の学びを支援するための工夫は、【輸液管理の経験と観察指導の促し】、【事前の学修内容や到達目標の共通理解】、【体験機会の調整】を含む、9のカテゴリと23のサブカテゴリで構成された。【実習中の観察と援助への意識づけ】や【振り返りとフィードバック】により、学生が輸液管理を意識し主体的に取り組む姿勢が見られたとの記述が示された。

演習段階と実習段階

輸液管理技術習得の指導困難と輸液管理実施の指導困難の関連(Table11)

輸液管理技術習得の指導困難と輸液管理実施の指導困難の関連について、（ρ =0.393、$p < 0.01$)と弱い相関が示された。

考察

演習段階における指導困難と実施状況の関連および指導困難の関連要因

演習段階において仮説1「輸液刺入部の再現性の困難は、輸液管理技術習得の指導困難に関連する要因である」を設定した。分析の結果、輸液刺入部の再現性に関して約半数の教員が困難を感じており、この再現性が困難なほど、輸液管理技術習得の指導に困難を感じる傾向が認められた。自由記述においても【刺入部を再現したリアルな構造】【リアルな異常所見の再現】【援助を想定した滴下対応】など、リアルな教材を求める意見が挙げられた。

これは、教員は現状の教材では刺入部の観察や判断に必要なリアリティが不足していると認識しており、再現性の困難が指導困難の関連要因になっている可能性があると考えられる。この結果は、輸液管理を行う援助場面は実像が伴わずイメージ化が困難であるため、実習での経験に委ねてしまう傾向がある[21]とした先行研究の指摘とも整合する。したがって、仮説1は支持され、教材の再現性を向上させることが、輸液管理技術習得の指導困難を軽減する可能性がある。

仮説2として、「輸液管理技術習得の指導困難は、学生の輸液管理技術項目実施状況と関連する」と設定した。分析の結果、多くの技術項目において教員が指導困難を感じるほど学生の実施状況が低い傾向が示された。「滴下速度の観察」や「滴下数の調整」など、教員が「助言や促しがあれば実施可能」と認識している項目でも指導困難との関連が認められた。このことは、根拠理解や状況判断を含めた技術習得の指導が困難にあると考える。自由記述には、【状況に応じた対応の思考】として＜トラブル場面を通じた判断の促進＞、【発問やフ

ィードバックによる思考促進】として＜観察ポイントの確認＞など多様な工夫が挙げられ、教員が根拠や状況判断を伴う指導を意図的に行っていることがうかがわれた。

先行研究によれば、知識や技術の統合が不十分な学生は、手順通りには実施できても状況に判断や根拠に基づいた適切な実践が難しくなる[6]。一方、心理的･技術的な準備は学生の自己効力感を高め[27]、知識と技術の統合を促すことで学生の判断力が育成に資する[28]。以上より、輸液管理技術習得の指導困難と学生の輸液管理技術項目実施状況には関連が認められ、仮説 2 は支持された。教育的には、根拠理解や状況判断を重視した指導が望まれる。

以上の仮説 1 と 2 の結果より、輸液刺入部の再現性の困難が輸液管理技術習得の指導困難と関連し、指導困難を感じるほど輸液管理技術の実施状況が低かった。この状況は、臨床場面の再現性が不足すると学生の理解が抽象的になると指摘した先行研究[21]とも一致する。

この課題に対して、教員は【臨床的環境の再現】として、注射ラベルなど実物を用いた〈リアル感の追求〉、【知識と技術の定着】に向けた反復、【学習の深化と実践的思考の促進】としての発問・フィードバック等を用いており、学生の観察ポイントの意識化や主体的な取り組みが促されたとの記述が示された。これらの工夫は、根拠理解や状況判断の学習を支える可能性が示唆される。

しかし、これらの工夫だけでは、根本的な教材の再現性不足や実践的理解の課題を完全に解決するまでには至らないと考えられる。視認性の高い教材の開発やハイブリッドシミュレーションやVR(Virtual Reality)など臨床場面を想定した教育は、学生の判断力や自信の向上に資するとの報告もある[27,29]。したがって、段階的な導入が有用と考えられる。

実習段階における指導困難と実施状況の関連および指導困難の関連要因

実習段階では仮説 3 として、「学生の輸液管理見学・実施の機会は、輸液管理実施の指導困難に関連する要因である」を設定した。分析の結果、輸液管理の見学機会および実施機会が得られないほど、輸液管理実施の指導困難を感じる傾向が認められた。特に約 64%の教員が実施機会を得られないと回答しており、実施機会の不足が示唆された。実習施設に関連する背景として、【安全確保と倫理的配慮の優先】における＜リスクが大きく安全性の担保が困難＞、【実習施設との調整困難】などが挙げられた。また教育上の背景として、【学修段階を考慮した優先的指導】における＜看護過程の重視＞、【輸液管理に関する教育方針】における＜基本的ケア優先の教育的判断＞などが挙げられた。こうした背景が実施機会の不足を招き、指導困難につながっていると考える。先行研究でも実習で輸液管理を未経験のまま終える学生が多い[22~25]ことが報告されており、本研究の結果と一致している。したがって、仮説 3 は支持され、輸液管理の実施機会の不足が実習段階での指導困難の関連要因であることが示された。

仮説 4 として設定した「学生の輸液管理に関する知識・必要性の認識」が指導困難と関連するという点についても、輸液管理技術に関する知識や必要性の認識が低いほど、学生の輸液管理実施の指導困難を感じる可能性が示された。自由記述では、【学生の知識・技術不足による余裕のなさ】として＜輸液管理に関する知識・技術の不足＞、＜輸液管理への意識の低

さ＞など、具体的状況が挙げられた。これらは指導困難に関わる背景的な関連要因と示唆された。この結果は、看護学生の輸液管理に関する知識や技術不足が実習での輸液管理実施の困難につながることを指摘した先行研究[6,7,14]とも一致しており、教育内容の充実や実践的な学習機会の重要性を示唆している。したがって、仮説4は支持された。

仮説5として設定した「輸液管理実施の指導困難は学生の輸液管理実施状況（指導下）と関連する」という点についても、指導困難を感じるほど学生の輸液管理実施状況（指導下）が低い傾向がみられた。指導困難の理由では、【実施機会の限界】として＜輸液中の患者を受け持つ機会の不確実性＞、【輸液管理の指導時間と人的資源の不足】が示されている。すなわち、輸液管理を行う患者を、指導可能な状況で受け持つ機会を確保することが困難なうえ、十分な個別指導が困難になっていることが示された。こうした状況は、学生が輸液管理の十分な経験を積むことが難しく、技術への自信や安全意識の低下につながる[22-26]報告とも一致する。また、教員側の負担や人的資源不足により学生への個別指導やフィードバックが十分に行えない状況があることも、指導困難を増大させる重要な要素である[30,31]報告とも整合的である。以上のような指導環境の制約によって指導困難が生じ、その結果として学生の輸液管理技術習得状況の低下につながっている可能性が示唆された。したがって、仮説5も支持される。

以上、仮説3、4、5の結果より、実習段階における輸液管理実施の指導困難は、輸液管理の実施機会や学生の知識・必要性の認識不足などの関連要因によって生じ、指導下での実施状況に関連していることが明らかとなった。また、安全確保や倫理的配慮に伴う制約、指導時間や人的資源不足などがこれらの背景にある可能性が示唆された。まずは到達目標の共有により、観察視点と実施可否の基準を事前に整合させることが有用と考える。

演習段階の指導困難と実習段階の指導困難との関連

仮説6として設定した「演習段階の輸液管理技術習得の指導困難は、実習段階の輸液管理実施の指導困難と関連する」という点について分析した結果、演習段階で指導困難を感じるほど、実習段階でも輸液管理実施の指導困難を感じる傾向が示された。自由記述では、実習段階の指導困難の理由として【実習前に技術を高める演習が不十分】として＜教材の教育的適合性の不足＞＜教材・物品の整備困難＞などが挙げられた。こうした演習段階の課題が、実習段階での輸液管理実施の指導困難に波及し得ると考えられる。

こうした課題への対応として、教員は【実習前の輸液管理に関する意識づけと技術準備】として＜技術確認＞、【実習中の観察と援助への意識づけ】などの工夫を行っていた。さらに【輸液管理の経験と観察視点の促し】や【振り返りとフィードバック】を取り入れることで、学生が輸液管理を意識し主体的に取り組んだとの記載が複数みられた。これらの記述上の所見は、学内演習で形成された自信が実習での実施につながり得るとの報告[22]や、シミュレーション導入による能力・自信の向上を示す先行研究[11, 32]とも整合的である。学生が輸液管理を意識し主体的に取り組む様子が、自由記述からうかがえた。以上より、演習段階での技術習得や自信の育成は、実習段階における指導困難の軽減に資する可能性がある。

輸液管理技術教育における課題と具体的な対策

輸液管理技術教育における指導困難の背景として、輸液刺入部の再現性の困難、学生の状況判断に基づく輸液管理技術の未熟さ、輸液管理実施機会の不足、学生の知識や必要性の認識不足が示された。先行研究においては、シミュレーション教材の導入は学生の観察力や臨床判断力向上に有効である[33,34]ことが報告され、発問やフィードバックなど実践的な教育法の重要性も示されている[28,29]。具体的な対策として、演習段階では視認性の高い教材やVR教材などを導入し、シナリオベースの教育の導入など、実践的な教育方法が有効と考える。

実習段階では、施設との調整や指導体制の強化が重要である。演習段階における学生の実施状況や指導マニュアルなどを臨地実習指導者と共有することで、学生の状況が明確になり、指導が効果的になる可能性がある。実習施設とのパートナーシップが学生の技術習得を支援することも報告されており[35]、継続的な連携が求められる。また、戦略的な指導によって学生の臨床推論能力や実践的洞察力を高めること[36]、心理的・技術的準備による看護介入の質を向上させること[37,38]も効果的である。

これらの課題と対策から、実習で指導の下で輸液管理実施に向け、視認性の高い教材と段階的な判断力の育成を重視した演習プログラムの整備が必要である。

結論

輸液管理技術の指導困難の関連要因として、演習段階の輸液刺入部の再現性困難、輸液管理実施機会の制約、学生の知識・認識不足が示唆された。演習段階での指導困難は学生の輸液管理技術項目実施状況と関連し、実習段階での指導困難は指導下の輸液管理実施状況と関連していた。さらに、演習段階の指導困難と実習段階の指導困難にも関連性がみられた。

研究の限界と今後の課題

本研究は、輸液管理の卒業時到達度が「指導の下で実施できる」に改正されたことを踏まえ、2023 年度に教員を対象として実施した。回答は新しい到達目標への対応を必ずしも反映していない。また、本研究は同一時点の教員自己報告に依拠しており、測定法由来(同一情報源)バイアスの可能性がある。また、学校 ID を収集していないため教育機関レベルの調整は行っておらず、同一機関からの複数回答の影響を完全には排除できない。加えて、回答率が30%にとどまっていることから、得られた結果の一般化には限界がある。

今後は、対象者数を拡大し、教員・学生双方からデータを収集して、多角的に検討し、一般化可能性を検証する必要がある。さらに、視認性の高い教材整備と判断力育成を重視した段階的演習の設計・試行に加え、到達目標の共有等を含む実習施設との連携運用を検討し、「実習で指導の下で実施」達成への有効性を検証する。

謝辞

本研究の実施にあたり、ご多忙の中アンケート調査にご協力くださった全国の看護基礎教育機関の教員の皆様に、心より感謝申し上げます。

引用文献

1.Ministry of Health, Labour and Welfare. Guidelines for the Operation of Training Schools for Public Health Nurses, Midwives, and Nurses; 2023 (in Japanese).<https://www.mhlw.go.jp/kango_kyouiku/_file/1.pdf>(Accessed April 23, 2025)
2.Nursing and Midwifery Board of Australia. Registered nurse standards for practice; 2016. <https://www.nursingmidwiferyboard.gov.au/codes-guidelines-statements/professional-standards/registered-nurse-standards-for-practice.aspx> (Accessed April 23, 2025)
3.American Association of Colleges of Nursing. The Essentials: Core Competencies for Professional Nursing Education; 2021. <https://www.aacnnursing.org/Portals/0/PDFs/Publications/Essentials-2021.pdf> (Accessed April 23, 2025)
4.Nursing and Midwifery Council. Standards of proficiency for registered nurses; 2018. <https://www.nmc.org.uk/standards/standards-for-nurses/standards-of-proficiency-for-registered-nurses/> (Accessed April 23, 2025)
5.Terry VR, Moloney C, Bowtell L, Terry PC. Online intravenous pump emulator: As effective as face-to-face simulation for training nursing students. Nurse Educ Today 2016;40:198-203.
6.Hernon O, McSharry E, Simpkin AJ, MacLaren I, Carr PJ. Evaluating nursing students' venipuncture and peripheral intravenous cannulation knowledge, attitude, and performance: a two-phase evaluation study. J Infus Nurs 2024;47:108-19.
7.Simonetti V, Comparcini D, Miniscalco D, Tirabassi R, Di Giovanni P, Cicolini G. Assessing nursing students' knowledge of evidence-based guidelines on the management of peripheral venous catheters: A multicentre cross-sectional study. Nurse Educ Today 2019;73:77-82.
8.Vandenhouten CL, Owens AK, Hunter MR, Raynak A. Peripheral intravenous education in North American nursing schools: a call to action. J Nurs Educ 2020;59:493-500.
9.North Central Missouri College. Nursing Student Handbook 2024-2025; 2024. <https://www.ncmissouri.edu/academics/wp-content/uploads/sites/2/2018/08/Nursing-Student-Handbook.pdf> (Accessed April 23, 2025)

10. Huang J, Liu X, Xu J, Ren L, Liu L, Jiang T, Huang M, Wu Z. Examining the effect of training with a teaching for understanding framework on intravenous therapy administration's knowledge, performance, and satisfaction of nursing students: a non-randomized controlled study. BMC Nurs 2024;23:104.
11. Lofton CM, Schmaldinst KE, Cozort RW. Implementation of a supervised focused infusion therapy clinical experience and its impact on confidence and competence. J Infus Nurs 2021;44:216-23.
12. Asadizaker M, Abedsaeedi Z, Abedi H, Alijanirenani H, Moradi M, Jahani S. Improvement of the first training for baccalaureate nursing students--a mutual approach. Glob J Health Sci 2015;7:79-92.
13. Sherriff K, Wallis M, Burston S. Medication calculation competencies for registered nurses: a literature review. Australian Journal of Advanced Nursing 2011;28:75-83.
14. Kaliyaperumal R, Jeyapaul S, Chellathurai A. Intravenous therapy: nursing students' knowledge and confidence. Int J Health Sci Res 2023;13:56-63.
15. Lee JS. Implementation and evaluation of a virtual reality simulation: intravenous injection training system. Int J Environ Res Public Health 2022;19:5439.
16. Ota Y, Aikawa G, Nishimura A, Kawashima T, Imanaka R, Sakuramoto H. Effects of educational methods using extended reality on pre-registration nursing students' knowledge, skill, confidence, and satisfaction: a systematic review and meta-analysis. Nurse Educ Today 2024;141:106313.
17. Ishiguro C, Tochikawa A, Kawamura R, Taguchi E, Ishihara K, Yamamoto Y, Ishida S, Taniguchi J, Wada Y, Higashino T. On-campus training using high- and intermediate-fidelity simulations to promote clinical practice skills of nursing students. Journal of Japanese Red Cross Toyota College of Nursing 2023;18:15-21 (in Japanese).
18. Masuda M, Sugiyama F, Yagi M. A status survey of simulation-based learning in Japanese undergraduate nursing education. Annual report of the Murata Science Foundation 2020;34:664-6 (in Japanese).
19. Sakuma R, Imatuji Y, Tsuruoka Y, Yoshimura C, Tsukahara H. Kango gakusei no iryō anzen kakunin kōdō no kōjō no tame no shimyurēshon enshū kyōzai no kaihatsu to hyōka (Development and evaluation of simulation-based training material to improve nursing students' safety confirmation behavior).Nihon Kango Gakkai Ronbunshū Kango Kyōiku 2010;40:122-4 (in Japanese).
20. Anazawa S, Yoshimitsu S, Matsuyama T. Experience of students who carried out an assignment to exchange sleepwear of a patient undergoing drip infusion, by using

prepared teaching material that allows dripping of an infusion fluid. Journal of Japanese Society of Nursing Science for National Health Services 2009;5:2-10 (in Japanese).
21. Nakayama K, Takahashi A, Kimura N, Matsumoto S, Chikugo Y, Aida M, Nakazawa Y, Fuse H. Kiso kango-gaku ryōiki ni okeru jōmyaku nai chūsha gijutsu no kyōiku hōhō no kentō (A study on teaching methods of intravenous injection techniques in basic nursing education).
Bulletin, Saitama Prefectural University Junior College 2004;6:89-96 (in Japanese).
22. Harada M, Nitta J, Osanai C, Urushizaka M, Nakamura R, Kimura K, Murata C.
The current state of and future problems involving the practice and acquisition of nursing skills in adult Nursing Clinical Training: Clarification of the characteristics of chronic/perioperative phase training and Moves Toward more complete in-class exercise . Bulletin of Faculty of Nursing Hirosaki Gakuin University 2009;4:11-24 (in Japanese).
23. Ishimitsu F, Furuya T, Kuchimoto S, Hayashi M, Takeuchi K, Ito M, Arai K.
Seijin kango-gaku jisshū ni okeru gakusei no kango gijutsu keiken no jittai (Experience of nursing techniques among students in adult nursing clinical practice). Mejiro Journal of Health Care Sciences 2010;3:75-9 (in Japanese).
24. Miura Y, Yamanaka M, Hiraga M, Morioka H, Nakamoto A, Fujiwara N.
Seijin kango-gaku jisshū ni okeru kango gijutsu keiken no jittai (Experience of nursing procedures in the clinical practice of adult nursing). The Journal of Senri Kinran University 2016;13:125-33 (in Japanese).
25. Ogiwara M, Nitta J, Saito T, Watanabe K, Kitabayashi M, Isozaki F, Miyahori M.
Four-year research on nursing-skill experiences in adult nursing clinical practicum of the students of College A. Journal of the Japanese Red Cross Akita College of Nursing and the Japanese Red Cross Junior College of Akita 2017;22:47-56 (in Japanese).
26. Suenaga M, Takenobu Y.
The Nursing Skills, What the Students Finishing Nursing College Are Afraid That They Didn't Study Enough.
Journal of Shonan Junior College 2010;21:49-57 (in Japanese).
27. Uzelli Yilmaz D, Sari D. Examining the effect of simulation-based learning on intravenous therapy administration' knowledge, performance, and clinical assessment skills of first-year nursing students. Nurse Educ Today 2021;102:104924.

28. Ahmadi S, Abdi A, Nazarianpirdosti M, Rajati F, Rahmati M, Abdi A. Challenges of clinical nursing training through internship approach: a qualitative study. J Multidiscip Healthc 2020;13:891-900.
29. Chang YY, Chao LF, Chang W, Lin CM, Lee YH, Latimer A, Chung ML. Impact of an immersive virtual reality simulator education program on nursing students' intravenous injection administration: a mixed methods study. Nurse Educ Today 2024;132:106002.
30. Dağ GS, Kılıç HF, Görgülü RS. Difficulties in clinical nursing education: views of nurse instructors. Int Arch Nurs Health Care 2019;5:114.
31. Benny J, Porter JE, Joseph B. A systematic review of preceptor's experience in supervising undergraduate nursing students: lessons learned for mental health nursing. Nurs Open 2023;10:2003-14.
32. Marchionni C, Connolly M, Gauthier M, Lavoie-Tremblay M. Innovative approaches to teaching vascular access to nursing students in the COVID-19 era. Br J Nurs 2021;30(14):S34-41.
33. Oh PJ, Jeon KD, Koh MS. The effects of simulation-based learning using standardized patients in nursing students: a meta-analysis. Nurse Educ Today 2015;35:e6-15.
34. Oda C, Kato M. Examining the "Virtual Reality Critical Care Nursing Kanzu Approach" for Clinical Assessment. Journal for the study of cooperation in education 2023;18:75-92 (in Japanese).
35. Bvumbwe T. Enhancing nursing education via academic-clinical partnership: an integrative review. Int J Nurs Sci 2016;3:314-22.
36. Lazarus J. Precepting 101: teaching strategies and tips for success for preceptors. J Midwifery Womens Health 2016;61(S1):11-21.
37. Avşar G, Yıldız G, Özcan S. A scale of readiness for clinical practice: a validity and reliability study for nursing students. Teach Learn Nurs 2024;19:e412-e9.
38. Hasanah O, Haryati RTS, Wanda D, Agustini N, Masfuri M. Readiness of nursing students for clinical practice: a literature review. Healthc Low Resour Settings 2024;12:13014.

Table 1. Survey content—Skills training phase

Survey items (variables)	Measure description	Response format
Difficulty replicating infusion insertion sites	Degree of difficulty in replicating the infusion insertion site using training materials/models	5-point scale: 5 = difficult; 1 = not difficult
Instructional difficulty in facilitating infusion-management skill acquisition	Degree of instructional difficulty in advancing students to the "able to perform under supervision (using a mannequin or peer practice)" level	5-point scale: 5 = difficult; 1 = not difficult
Students' ability to implement infusion-management skill items (16 items)	Extent to which students can currently apply the 16 infusion-management skill items defined with reference to textbooks	5-point scale: 5 = can perform independently; 1 = unable even with prompting
Educational materials required for infusion-management skill acquisition	Educational materials considered necessary for infusion-management skill acquisition	Free-form response
Instructional strategies for infusion-management skill acquisition	Instructional strategies used to facilitate infusion-management skill acquisition	Free-form response

Table 2. Survey content—Clinical practicum phase

Survey items (variables)	Measure description	Response format
Students' opportunities to observe and perform infusion management	Adequacy of students' opportunities to observe and perform infusion management during the clinical practicum	5-point scale: 5 = ample opportunities; 1 = no opportunities
Students' knowledge of infusion management and recognition of its necessity	Degree to which students are perceived to possess knowledge of infusion management and to recognize its necessity	5-point scale: 5 = high; 1 = low
Instructional difficulty in implementing infusion management	Degree of instructional difficulty in guiding students to implement infusion management under supervision	5-point scale: 5 = difficult; 1 = not difficult
Proportion of students able to perform infusion management (under supervision)	Percentage of students who can perform infusion management under supervision at the time of the respondent's practicum supervision	5-point scale (percentage bands): 5 ≈ 100%; 1 = almost none
Reasons for instructional difficulty in implementing infusion management	Reasons for the above instructional difficulties in implementing infusion management	Free-form response
Instructional strategies to facilitate implementation of infusion management	Instructional strategies to enable students to implement infusion management under supervision	Free-form response

Table 3. Basic characteristics of participants and institutions (n = 375)

Item		n	(%)
Basic Attributes of Participants			
Age Group	20s–30s	59	(15.7)
	40s	155	(41.3)
	50s	135	(36.0)
	60s	26	(6.9)
		Mean age ± SD = 47.9 ± 8.1 years	
Gender	Male	57	(15.2)
	Female	312	(83.2)
	No response	6	(1.6)
Years of Experience as a Nursing Educator	Less than 5 years	74	(19.7)
	5–10 years	115	(30.7)
	10–15 years	75	(20.0)
	15–20 years	48	(12.8)
	20+ years	63	(16.8)
		Mean years ± SD = 11.3 ± 7.5 years	
Area of Expertise	Basic Nursing	172	(45.9)
	Adult Nursing	161	(42.9)
	Integrated Nursing and Practice	8	(2.1)
	Home Care Nursing	9	(2.4)
	Pediatric Nursing	8	(2.1)
	Psychiatric Nursing	4	(1.1)
	Maternal Nursing	6	(1.6)
	Geriatric Nursing	7	(1.9)
Basic Attributes of Organizations			
Type of School	University	122	(32.5)
	Junior College	8	(2.1)
	College of Nursing	3	(0.8)
	Vocational School	232	(61.9)
	Other/No response	10	(2.6)
Relationship with clinical practicum facilities (multiple responses allowed)	Same organization	176	
	Educational/research collaboration	28	
	Commissioned as clinical practicum facilities	239	
	Undetermined	4	

Table 4. Association between difficulty replicating infusion insertion sites and instructional difficulty in facilitating infusion-management skill acquisition

	Difficulty replicating infusion insertion sites
Instructional difficulty in facilitating infusion-management skill acquisition	.399**

Spearman's rank correlation coefficient, ρ; $p < 0.01$; n = 299

Table 5. Association between instructional difficulty in facilitating infusion-management skill acquisition and students' ability to implement infusion-management skill items

	Instructional difficulty in facilitating infusion-management skill acquisition
Observation of drip rate	-.329**
Adjustment of drip rate	-.301**
Observation of remaining infusion volume	-.256**
Confirmation of fixation of the infusion needle/cannula	-.247**
Observation of skin abnormalities around the insertion site	-.217**
Observation of blood backflow in the infusion line	-.164**
Confirmation of dressing adhesion at the insertion site	-.223**
Checking for looseness at connections	-.239**
Checking for kinking or compression of the infusion line	-.239**
Checking for pulling or tension on the infusion line	-.197**
Confirmation of the position of the infusion line	-.212**
Checking for air entry in the infusion line	-.248**
Observation of the patient's overall condition	-.165**
Assistance with safe and comfortable positioning during infusion	-.200**
Reporting related to infusion management	-.231**
Appropriate response when abnormalities are detected	-.161**

Spearman's rank correlation coefficient ρ; $p < 0.01$; n = 305

Table 6. Educational materials required for infusion-management skill acquisition

Category	Sub-category
Realistic structure simulating the puncture site	Materials for confirming blood backflow<br>Improved models for observing puncture site and drip<br>Materials for observing infusion puncture sites<br>Realistic representation of infusion puncture sites
Reproduction of abnormal findings	Materials that simulate complications<br>Models that allow observation of abnormal puncture sites<br>Models that realistically replicate puncture site abnormalities
Drip management with support	Materials allowing observation of drips<br>Materials that enable observation and assistance during dripping
Handling of sequential procedures	Models allowing IV insertion and dripping<br>Models allowing venipuncture and confirmation of blood backflow<br>Models with tape fixation capability for puncture sites<br>Materials for practicing IV puncture<br>Pediatric practice models for infusion
Materials with adhesion and fixation	Models with improved tape adhesion
Cost-effectiveness	Affordable training materials<br>Low-cost and effective materials
Ease of maintenance	Models with replaceable or reusable parts<br>Washable and detachable models<br>Easy-to-maintain materials<br>Training kits for infusion management
ICT-based learning support	ICT-based individualized learning materials<br>Audiovisual learning materials

Table 7. Instructional strategies for infusion-management skill acquisition

Category	Sub-category
Pre-learning to confirm knowledge and skills	Pre-learning using audiovisual materials<br>Pre-learning for technical training<br>Confirmation of knowledge based on rationale
Supervisory framework ensuring student safety and reassurance	Supervisory framework ensuring student safety<br>Instruction system designed to reduce fear and anxiety
Instructors acting as simulated patients	Instructors acting as simulated patients
Reproduction of clinical environment	Pursuit of realism<br>Use of real or realistic materials
Promoting critical thinking through questioning and feedback	Feedback from instructors and simulated patients<br>Confirmation of observation points
Promoting deep and practical thinking	Instruction linked to skills and assessment<br>Opportunities for reflection through scenario presentation<br>Stepwise acquisition of knowledge and skills
Encouraging situational responses	Encouraging judgment in problematic scenarios<br>Experience of care involving patient movement
Experiencing specific roles	Experience of being a patient<br>Understanding nurses' perspectives
Acquisition of knowledge and skills	Acquisition of appropriate techniques<br>Learning methods linked to knowledge and skills<br>Setting up environments that promote active knowledge and skills<br>Use of skill checklists and tests<br>Practice of sequential tasks from drip start to finish
Integration and application of knowledge	Connection to the curriculum<br>Pre-graduation skills training<br>Opportunities to accumulate experience
Pump management	Instruction on operation and management of pumps

Table 8. Associations between instructional difficulty in implementing infusion management and related factors

	Instructional difficulty in implementing infusion management
Students' opportunities to observe infusion management	-.260** (n =306)
Students' opportunities to perform infusion management	-.219** (n =303)
Students' knowledge of infusion management	-.207** (n =305)
Students' recognition of the necessity of infusion management	-.211** (n =305)
Proportion of students able to perform infusion management (under supervision)	-.429** (n =277)

Spearman's rank correlation coefficient, ρ; $p < 0.01$; pairwise deletion; n varies by pair

Table 9. Reasons for instructional difficulty in implementing infusion management

Category	Sub-category
Insufficient skills enhancement prior to the clinical practicum	Lack of educational suitability of instructional materials<br>Difficulty in preparing teaching materials and equipment<br>Emphasis on other educational content
Prioritized instruction based on students' learning phase	Emphasis on the nursing process<br>Educational phase of the fundamental nursing practicum
Educational policy regarding infusion management	Educational decision to prioritize basic care<br>Educational decision considering institutional and ethical constraints<br>Outside the scope of educational achievement goals
Limited capacity due to lack of knowledge and skills	Students' low awareness of infusion management<br>Students' low prioritization of infusion management<br>Insufficient student knowledge and skills regarding infusion management<br>Students are preoccupied with patient assessment and care
Lack of time and human resources for teaching infusion management	Difficulty in securing instructional time<br>Shortage of human resources
Priority given to safety and ethical considerations	High risk makes it difficult to ensure safety<br>Ethical considerations for patients take priority
Coordination difficulties with clinical practicum sites	Difficulties coordinating with clinical practicum sites<br>Adherence to facility policies
Limited opportunities for hands-on practice	Uncertainty in opportunities to care for patients receiving infusions<br>Lack of opportunities to gain experience during the practicum period<br>Restrictions on the clinical practicum due to COVID-19
Observation only (no hands-on practice)	Observation only; hands-on practice not allowed

Table 10. Instructional strategies to facilitate implementation of infusion management

Category	Sub-category
Pre-practicum awareness-raising and skills preparation for infusion management	Explanation of infusion management during orientation<br>Pre-practicum skills check<br>Pre-practicum preparatory learning<br>Instruction to integrate into care planning<br>Promotion of active learning using checklists
Promoting awareness of observation and assistance during the clinical practicum	Encouraging focus on infusion before observation or assistance<br>Promotion of active participation
Promoting experience and observation perspectives in infusion management	Thorough instruction in observation and drip rate calculation<br>Instruction to raise awareness of infusion content and observation perspectives
Promoting experience within the feasible scope during the clinical practicum	Awareness-building of drip rate calculation as part of infusion management<br>Instruction on observation as part of infusion management<br>Performing infusion management together with the instructor<br>Opportunities to experience infusion management outside assigned patients
Reflection and feedback	Post-observation reflection and prediction<br>Promoting understanding through timely reflection<br>On-campus practice based on knowledge and experience gained during the clinical practicum
Explanation provided by the instructor	Requesting explanations or guidance from instructors<br>Promoting visualization of potential complications
Shared understanding of pre-practicum training content and learning objectives	Understanding of on-campus skills training content<br>Achieving a shared understanding of pre-practicum learning content and graduation-level learning objectives through coordination meetings<br>Coordinating opportunities for hands-on practice during the clinical practicum through meetings
Adjustment of experiential opportunities	Adjusting experiential opportunities related to infusion management
Adjustment of observation opportunities	Adjustment of observation opportunities

Table 11. Association between instructional difficulty in facilitating infusion-management skill acquisition (skills training phase) and instructional difficulty in implementing infusion management (clinical practicum phase)

	Instructional difficulty in implementing infusion management
Instructional difficulty in facilitating infusion-management skill acquisition	$.393^{**}$

Spearman's rank correlation coefficient ρ; $p < 0.01$; n = 303

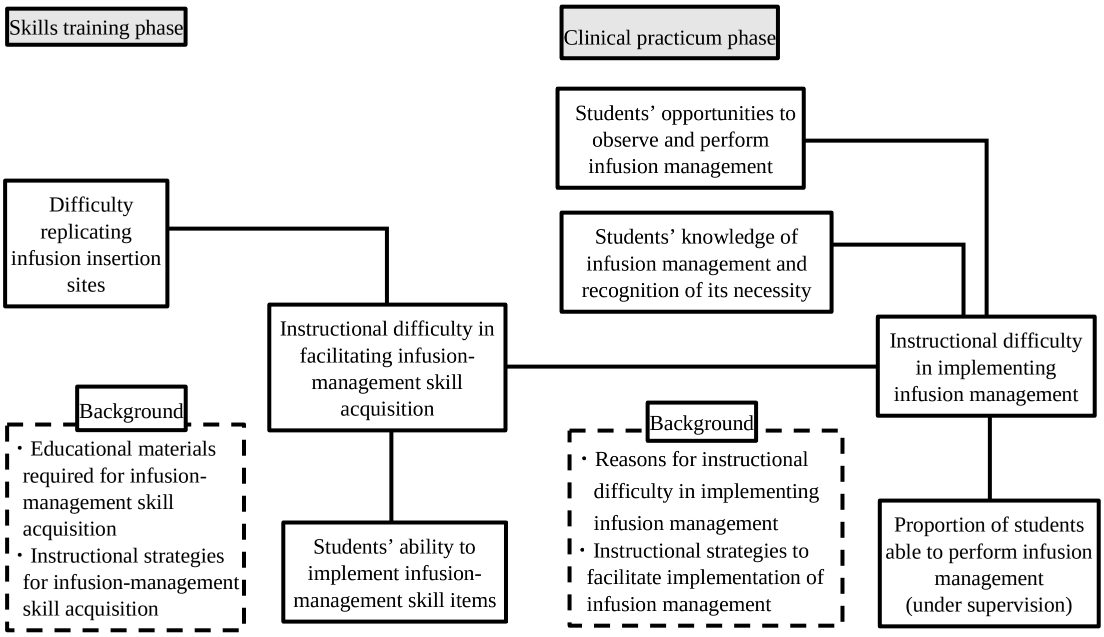

Figure 1. Conceptual framework of factors associated with the relationship between educators' instructional difficulty and students' implementation ability in infusion management

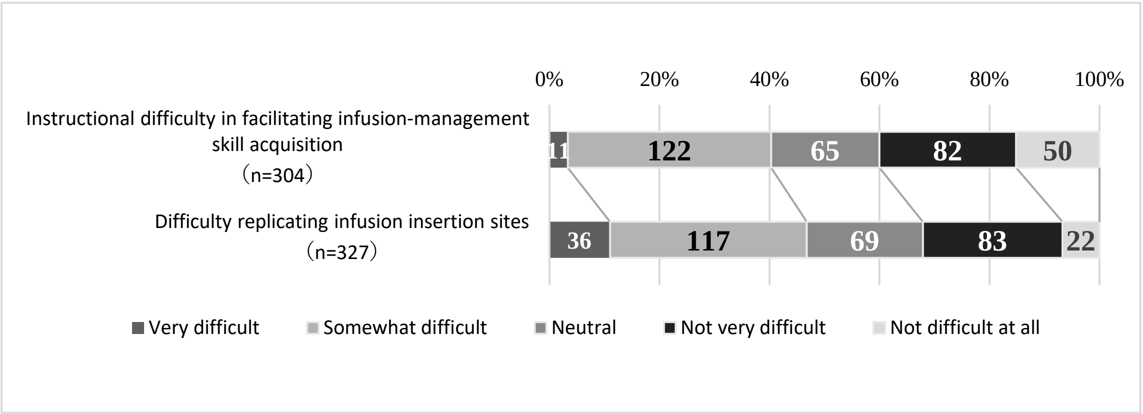

Figure 2. Difficulties in infusion insertion-site replication and infusion-management skill acquisition

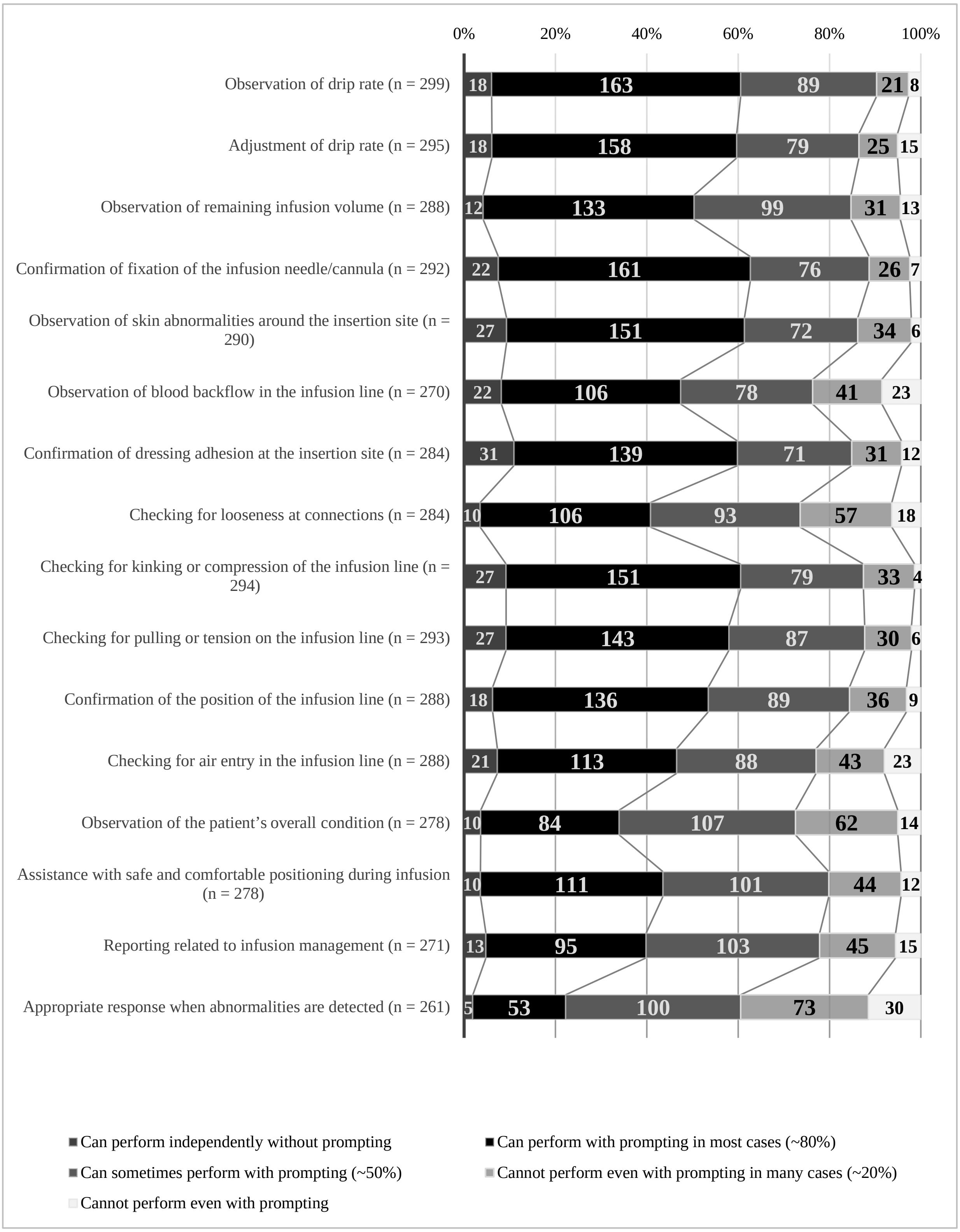

Figure 3 Students' ability to implement infusion-management skill items

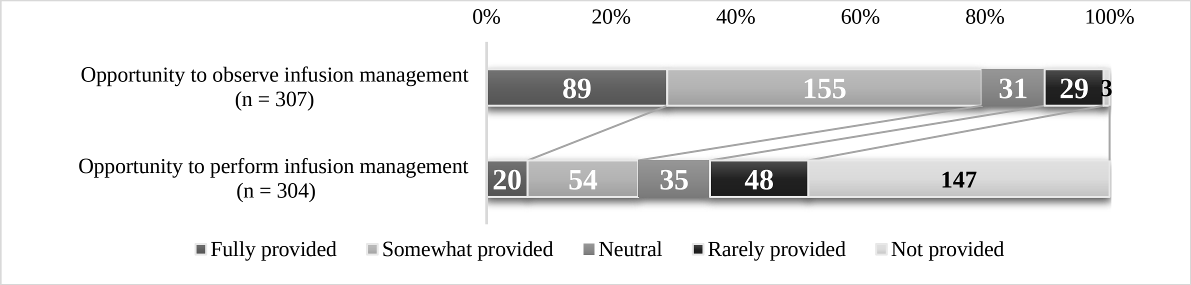

Figure 4 Students' opportunities to observe and perform infusion management

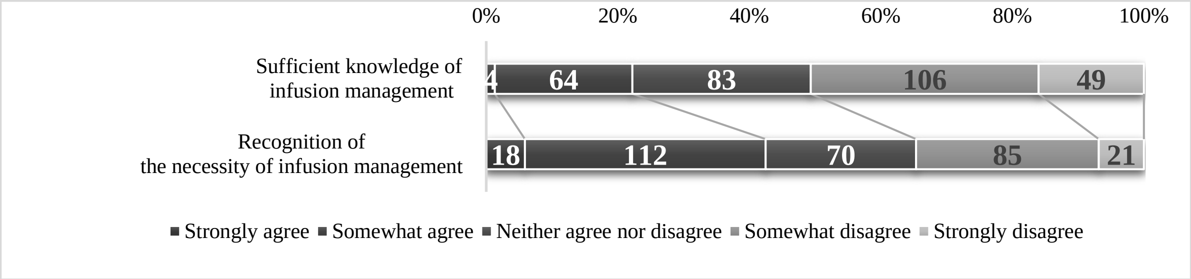

Figure 5 Students' knowledge of infusion management and recognition of its necessity n=306

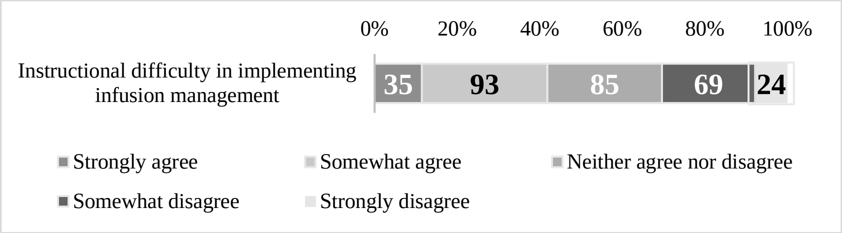

Figure 6 Instructional difficulty in implementing infusion management n=306

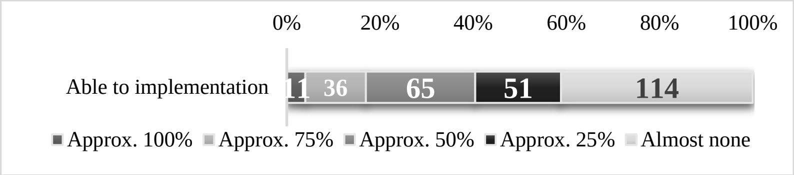

Figure 7 Proportion of students' able to implementation infusion management (under supervision) n＝277
